# Water Pharmacophore: Designing Ligands using Molecular Dynamics Simulations with Water

**DOI:** 10.1038/s41598-018-28546-z

**Published:** 2018-07-10

**Authors:** Sang Won Jung, Minsup Kim, Steven Ramsey, Tom Kurtzman, Art E. Cho

**Affiliations:** 1 Department of Bioinformatics, Korea University, Sejong, 30019 Korea; 2 Department of Chemistry, Lehman College, Bronx, New York 10468 USA; 3 Ph.D. Program in Chemistry, The Graduate Center of the City University of New York, 365 5th Avenue, New York, New York 10016 USA; 4 Ph.D. Program in Biochemistry, The Graduate Center of the City University of New York, 365 5th Avenue, New York, New York 10016 USA; 5 Present Address: Center for Supercomputing and Big Data, DGIST, Daegu, 42988 Korea

## Abstract

In this study, we demonstrate a method to construct a water-based pharmacophore model which can be utilized in the absence of known ligands. This method utilizes waters found in the binding pocket, sampled through molecular dynamics. Screening of compound databases against this water-based pharmacophore model reveals that this approach can successfully identify known binders to a target protein. The method was tested by enrichment studies of 7 therapeutically important targets and compared favourably to screening-by-docking with Glide. Our results suggest that even without experimentally known binders, pharmacophore models can be generated using molecular dynamics with waters and used for virtual screening.

## Introduction

**Figure 1:**
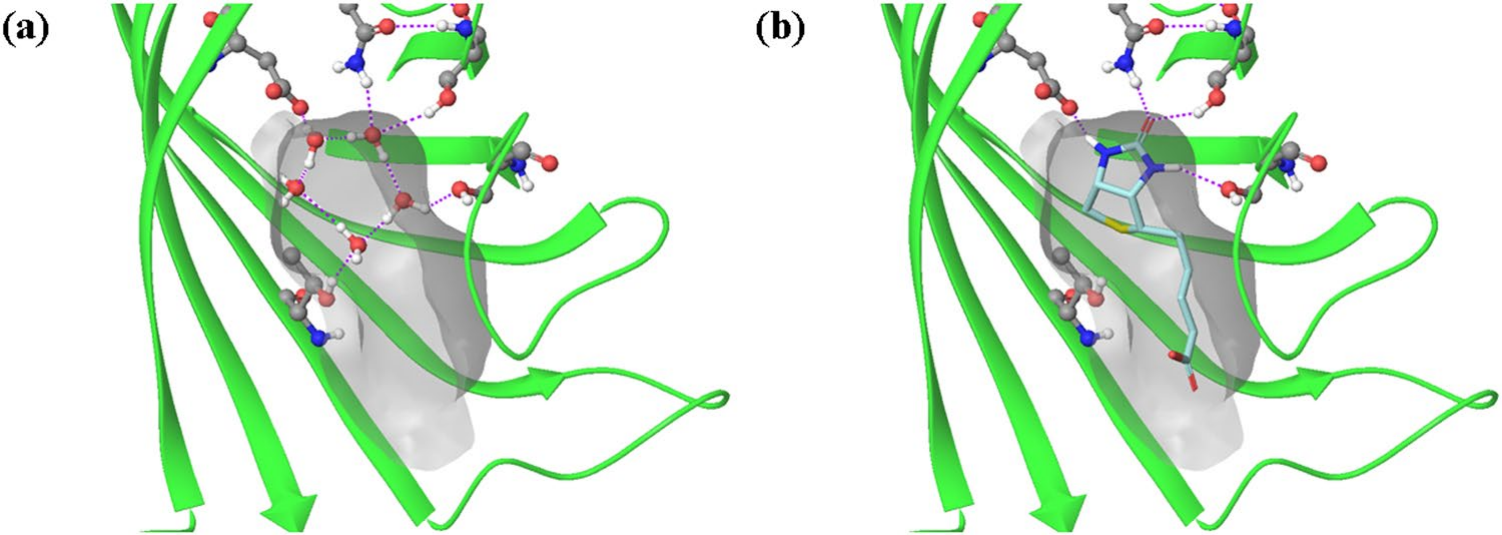
(**a**) Water and (**b**) biotin in the active site of streptavidin. Note how the water molecules and the ligand make similar contacts to the protein surface.

It is a fundamental tenet of drug design that, in order to potentially bind with high affinity to a given protein, a ligand must be complementary to that protein surface by donating and accepting hydrogen bonds and making hydrophobic contacts where appropriate^1,2^. Water molecules solvating a protein surface are complementary to the surface in that they donate and accept hydrogen bonds where appropriate and make corresponding van der Waals contacts with hydrophobic patches of the surface (Fig. 1)^3,4^. In this sense, water on a protein surface mimics the key interactions that a ligand must have in order to bind with high affinity to a targeted protein.

The construction of a pharmacophore is aimed at distilling the essential features that ligands must have to bind to a target^5,6^. The fact that water, when solvating a binding site, has many of these features suggests that a pharmacophore could be constructed based on an analysis of the hydration of an active site alone.

Here, we introduce a pharmacophore generation method that is based solely on the information provided from analysis of water interactions with the protein surface as observed in molecular dynamics simulations. As an initial application this approach was explored against the streptavidin-biotin system which is known to have highly ordered waters that correlate with ligand interactions. In further study, water-based pharmacophore models were constructed and screened against 7 pharmaceutically relevant target proteins from DUD, with use of their test sets^7^. In addition, we compare these results to screening of the same chemical library using conventional docking methodology with Glide^8^.

In this paper, we first detail the methodology used to construct the water-based pharmacophores, which we call simply water pharmacophore (WP), starting from the molecular dynamics simulations on a target protein. Then, we present the resulting pharmacophores for 7 different proteins, and demonstrate the results of screening and enrichment studies (Table 1). Finally, we discuss the results and suggest how the method can be implemented as either a stand-alone technique or can be incorporated into existing drug-discovery schemes in order to improve their efficacy.

**Table 1:** Targets and DUD sets for enrichment studies.

Target	Number of actives	Number of decoys	PDB ID
Acetylcholinesterase (AChE)	107	3892	4EY7
Androgen receptor (AR)	79	2854	1XQ2
Glucocorticoid receptor (GR)	78	2947	1M2Z
Peroxisome proliferator-activated receptor gamma (PPARγ)	85	3127	1ZEO
Poly(ADP-ribose) polymerase (PARP)	35	1351	1EFY
Progesterone receptor (PR)	27	1041	1SR7
Retinoid X receptor alpha (RXRα)	20	750	1MVC

## Methods

### Structure selection and preparation

The X-ray crystal structures of 7 targets - Acetylcholinesterase (AChE)^9^, Androgen receptor (AR)^10^, Glucocorticoid receptor (GR)^11^, Peroxisome proliferator activated receptor gamma (PPARγ)^12^, Poly (ADP-ribose) polymerase (PARP)^13^, Progesterone receptor (PR)^14^, and Retinoid X receptor alpha (RXRα)^15^ - were retrieved from the Protein Data Bank (PDB)^16,17^. These 7 targets were selected for this study from the original DUD test set based on their enrichment results in that study, with most systems producing maximum enrichment factors (EF_max_) ~50 with one producing a high EF_max_ of >100 (RXRα) and a few producing low EF_max_ of ≤5 (AChE, PPARγ, and PR)^7^. These structures were prepared by protein preparation wizard (PPW) module^18^ of Schrödinger suite. PPW adds hydrogen and neutralizes side chains that are neither close to binding cavity nor involved in the formation of salt bridges. In the next step, water molecules are removed and hydrogen atoms are added to the structure, at the most likely positions of hydroxyl and thiol hydrogen atoms. Protonation states and tautomers of His residue and Chi “flip” assignment for Asn, Gln, and His residues are selected during this step as well. Finally, minimization was performed until the average RMSD of non-hydrogen atoms reaches 0.3 Å.

### Molecular dynamics simulations

Molecular dynamics simulations were performed using AMBER12 software^19,20^ with AMBER99SB force field^21^ and TIP3P water model^22^. Ligands were not simulated; however, protein bound conformations were restrained during sampling at 2.5 kcal/mol·Å^2^ so as to sample water which could be found in a bound state binding site. Protein systems were encased in a cubic of TIP3P water molecules in which each edge had a minimum distance of 10 Å from protein heavy atoms with cubic periodic conditions. First, 10,000 steps of minimization with 100 kcal/mol·Å^2^ restraints on all protein heavy atoms was performed. Then, the system was heated with the same restraints for 100 ps until the system reached 300 K in NPT conditions. Several simulation steps were then taken to slowly reduce the restraints on protein heavy atoms from 100 to 10 kcal/mol·Å^2^, these steps total 350 ps simulation time, again in NPT conditions. At this stage another minimization was performed to ensure no bad contacts occurred as the restraint strength has been dropped rapidly, this minimization is performed for 10,000 steps at 10 kcal/mol·Å^2^ restraints on heavy atoms. After this several NPT steps were performed to slowly release restraints from 10 kcal/mol·Å^2^ to 2.5 kcal/mol·Å^2^, these steps total 1.2 ns of simulation time. Finally, once the system is fully equilibrated, heated, and relaxed a production simulation was conducted under NVT conditions for 10 ns simulation time sampling every 1 ps with 2.5 kcal/mol·Å^2^ restraints and 8 Å non-bonding interaction cutoff. Data from this simulation was utilized to produce hydration sites. Langevin dynamics^23^ are utilized to stabilize simulation temperatures and NPT simulations were performed with the Berendsen barostat^24^.

### Method for constructing ligand-based pharmacophore

Ligand-based pharmacophore hypotheses were constructed in this study to serve as a visual comparison to the water-based pharmacophores for each system. We used PHASE module^25,26^ of Schrödinger suite with the native ligand poses to generate ligand-based pharmacophore models.

### Hydration site analysis

Hydration site analysis (HSA) was performed on waters near the binding site in each of the 7 pharmaceutically relevant targets (Tables S1–7). Water was determined to be in the binding pocket by proximity (within 5 Å) to the crystallized ligands. Hydration sites were selected by finding 1 Å spheres which contain the highest count of oxygen atom of water, these locales are recorded and all waters found within are excluded from becoming unique sites^4^. Selected sites are only considered for analysis if they contain twice bulk density under similar conditions (for 10,000 samples this implies greater than 2,800 oxygen atom of water found). These sites once generated were analyzed for thermodynamic qualities such as energy and hydrogen bonding characteristics each of which would be considered in determining water-based pharmacophore feature viability. Energy was determined by calculating system energy with and without the water found within the site, translational entropy through the histogram method, and orientational entropy through nearest neighbors.

### Pharmacophore feature assignment

Each identified hydration site could potentially be treated as a WP feature; however, pharmacophore screening tends to produce optimal results when 4–8 feature criteria are utilized. Subsequently, making each hydration site a feature would not be an ideal method. In an effort to design a technique which would identify key contacts without known binders, a method of screening and assigning features to hydration sites was designed so that the method could be utilized blind to a target and produce reasonable results. The detailed criteria are summarized in Fig. S1. This scheme was created by thermodynamic principles and then the detailed parameters were optimized through a trial and error selection trained against our test systems. Starting with differentiation by acceptor and donor ratio, each hydration site is filtered through series of criteria by energy values and acceptor/donor ratio. In case of aromatic ring and hydrophobic features, both hydrogen bond acceptor and donor ratio should be lower than 100%. Then, to differentiate the two features, SiteMap module^27,28^ of Schrödinger suite was utilized to determine the surface area of the hydrophobic surface A site with favorable enthalpy and acceptor/donor ratio was determined to either be a hydrogen bond donor or acceptor site based on its bonding characteristics. Both hydrogen bond donor and acceptor have enthalpic energies values less than −8.0 kcal/mol. In case of hydrogen bond donor, the acceptor and donor ratios should be less than 50% and more than 100%, respectively. Conversely, the acceptor and donor ratios should be more than 100% and less than 50%, respectively, in case of hydrogen bond acceptor. If there are 2 hydrogen bond acceptor features within 3.5 Å of each other, they are combined to be a negative feature. To differentiate between hydrogen bond donor and positive features the length of the bond was utilized where greater than 1.5 Å describes a donor feature and less than 1.5 Å describes a positive feature.

### Pharmacophore model generation

Pharmacophore features assigned as in the previous section were used to construct a pharmacophore model using the PHASE module of the Schrödinger suite. The detailed criteria are summarized in Fig. S2. This scheme was created to efficiently select essential features and exclude the redundant features, especially hydrophobic features. The algorithm reduces the pharmacophore features to a manageable number typically less than 8 as pharmacophore screening becomes inefficient with more than 8 features. In addition, the scheme optimizes the position of each assigned feature by hydrogen-bond-constrained docking or energy minimization. In case of negative feature, two pharmacophore features are combined to form one negative feature as previously described. A negative feature position is optimized via hydrogen-bond-constraint docking with acetic acid (CH_3_COO^−^) as ligand. In case of hydrogen bond donor feature, the position is adjusted via hydrogen-bond-constraint docking with water molecule (H_2_O) as ligand when the enthalpy energy is more than −9.0 kcal/mol. At last, in case of hydrophobic feature, the position is optimized via energy minimization while converting hydration site to methane molecule (CH_4_) when the enthalpy energy is more than −8.2 kcal/mol. Among the hydrophobic features, sites with favorable energies are excluded from the energy minimization. Consequently, a water-based pharmacophore model with a set of optimized pharmacophore features is constructed (Tables S1–7).

### Pharmacophore screening

In order to validate our WP model, the PHASE module of Schrödinger suite was utilized for screening against the pharmacophore models constructed for popular targets (Fig. S3). Conformers of the ligands were generated using the ConfGen module^29^ of Schrödinger suite for screening. The distance matching tolerance used was 1.5 Å and other parameters in the default settings so that hits were rejected if their alignment scores were greater than 1.2, their vector scores were less than −1.0, or volume scores were less than 0.0, or any combination thereof.

### Molecular docking

The set of ligands for 7 targets were docked to the binding site of each target proteins and by using the Glide module^8^ of Schrödinger suite. Glide uses grids for fast scoring; the grid-generation module was used to generate grids for the binding site of each target proteins. The van der Waals scaling and partial charge cutoff was set to 0.8 and 0.15, respectively. Next, the standard precision (SP) and extra precision (XP) mode of Glide was used to rank the ligands in the order of the lowest docking score.

### Enrichment factor analysis

Pharmacophore effectiveness was validated via enrichment factor assessment of DUD molecule sets^7^. Enrichment is the likelihood of selecting known active compounds from a portion of highly ranked hits during screening compared to randomly selected from the original molecule set. The enrichment factor is represented by

$${\rm{EF}}=({{\rm{Hits}}}_{{\rm{sampled}}}/{{\rm{N}}}_{{\rm{sampled}}})/({{\rm{Hits}}}_{{\rm{total}}}/{{\rm{N}}}_{{\rm{total}}})$$

where EF is enrichment factor, Hits_sampled_ is the number of true hits in the hit list, N_sampled_ is the number of compounds in the hit list, Hits_total_ is the number of hits in the full database, N_total_ is the number of compounds in the full database. In this research, we calculated enrichment factors for the actives found in the top scoring 1%, 5%, and 10% of the total compounds screened.

## Results and Discussion

**Figure 2:**
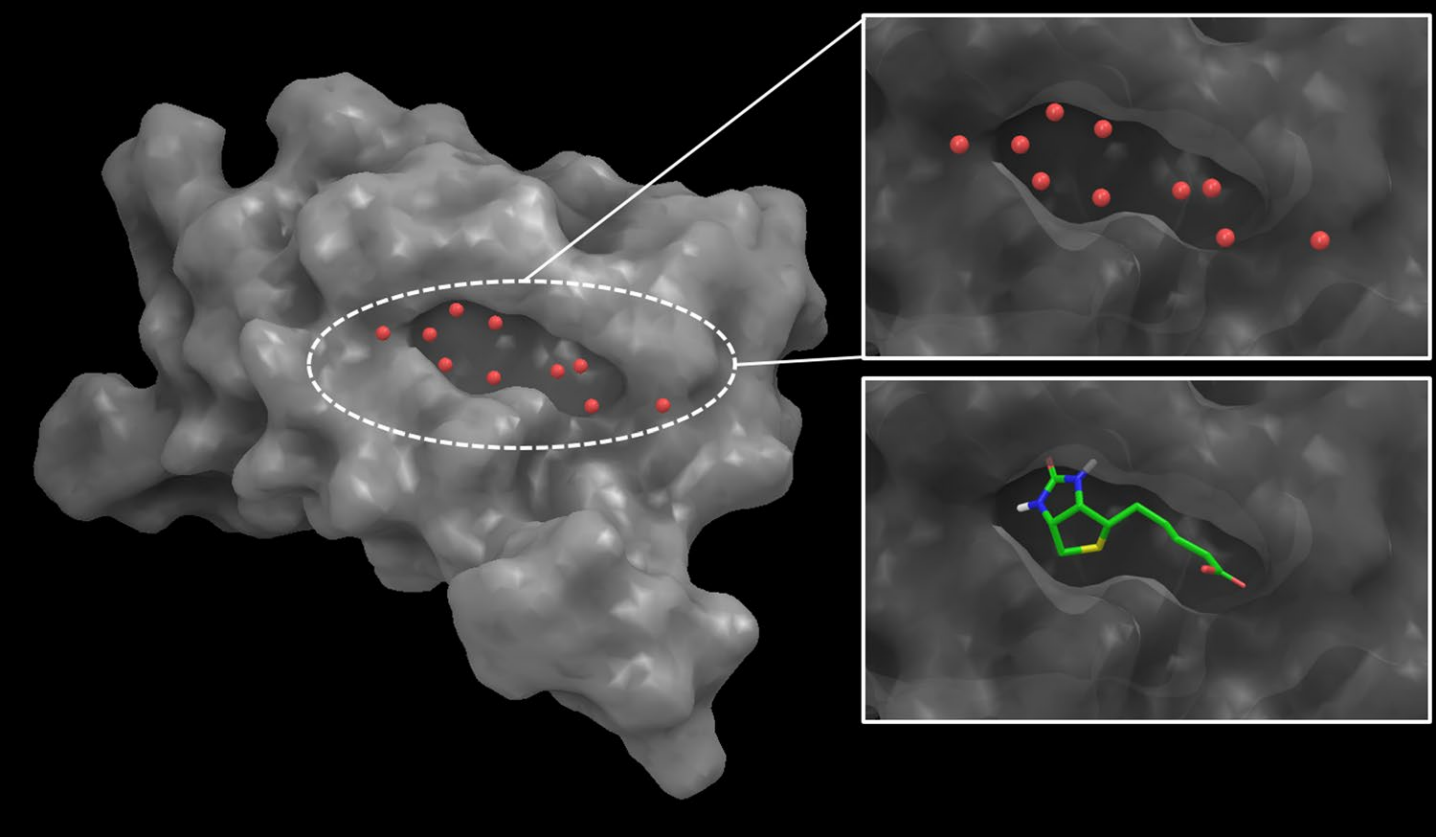
Hydration sites found in the binding site of streptavidin compared to the binding pose of its ligand biotin.

**Figure 3:**
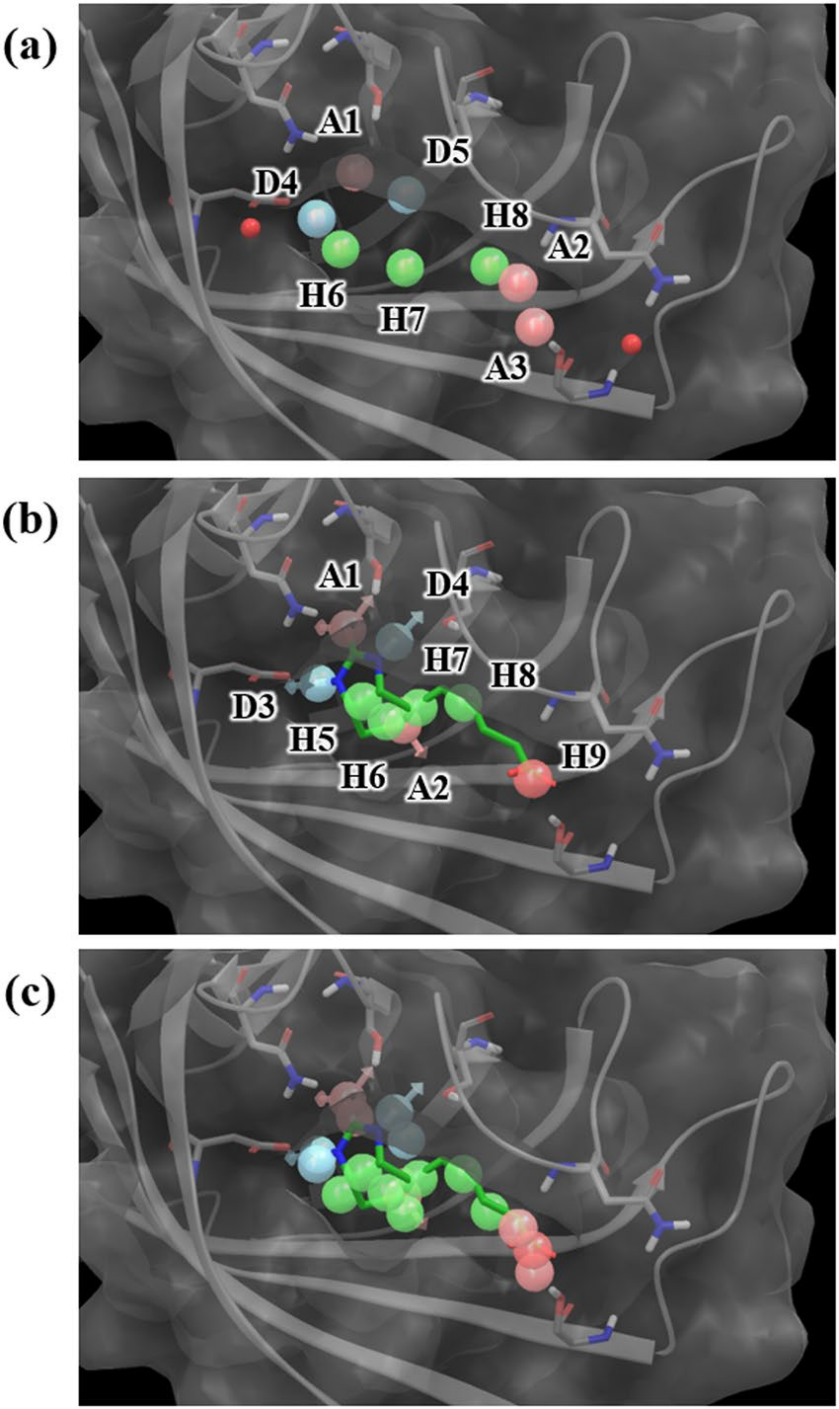
(**a**) Water- and (**b**) ligand-based pharmacophore model of biotin. (**c**) The two models overlaid in the binding site of streptavidin. The pharmacophore features are numbered to help identification of each site and colored as follows: hydrogen bond acceptor (A), pink; hydrogen bond donor (D), skyblue; hydrophobic (H), green; negative (N), red.

As shown in Fig. 2, 10 high density hydration sites that were generated from the MD simulations of the solvated streptavidin active site. Using our WP algorithm, we assigned pharmacophore feature to each of the 8 hydration sites out of 10, which were selected according to our algorithm. The resulting water-based pharmacophore is shown in Fig. 3a, alongside a ligand based pharmacophore hypothesis constructed off of biotin in Fig. 3b. An overlay of the water-based and ligand-based pharmacophores is shown in Fig. 3c. It appears that the ligand-based and water-based streptavidin pharmacophores overlap to a significant degree.

Using the same process, we then generated water-based pharmacophore and ligand-based pharmacophore for the 7 aforementioned targets.

### Enrichment study results

Tables 2 and 3 summarizes the results of enrichment study on the 7 targets.

**Table 2:** Enrichment analysis results using DUD sets for 7 targets by water-based pharmacophore screening.

Target	Number of Hydration sites	MODEL	EF 1%	EF 5%	EF 10%
AR*	11	DHHH	15.4	7.1	4.2
PR*	18	ADDHHH	21.6	8.2	4.4
RXRα	9	—	—	—	—
GR*	19	DHHH	19.4	6.7	3.3
PPARγ	25	DDHHHN	1.2	0.2	0.1
PARP*	36	ADDDR	22.6	5.2	2.6
AChE	43	DDDHHH	3.7	0.7	0.4

Enrichment factors are calculated for 1, 5, 10% of original data set. ‘−’Indicates that the model does not generate and evaluate the enrichment analysis. Entries marked with asterisk (*) are targets in which water-pharmacophore outperformed docking in EF 1%.

**Table 3:** Enrichment analysis results using DUD sets for 7 targets by Glide SP and XP docking.

Target	SP			XP		
	EF 1%	EF 5%	EF 10%	EF 1%	EF 5%	EF 10%
AR	11.5	6.6	5.3	10.2	6.6	4.8
PR	14.4	5.2	2.6	10.8	5.2	3.0
RXRα	24.1	14.8	8.5	19.3	11.8	8.0
GR	5.2	2.3	1.5	5.2	2.8	2.2
PPARγ	23.6	9.7	5.7	27.1	13.7	7.8
PARP	0.0	0.0	0.0	0.0	0.6	0.3
AChE	25.2	8.8	5.5	22.4	9.7	6.1

### Androgen receptor (AR)

**Figure 4:**
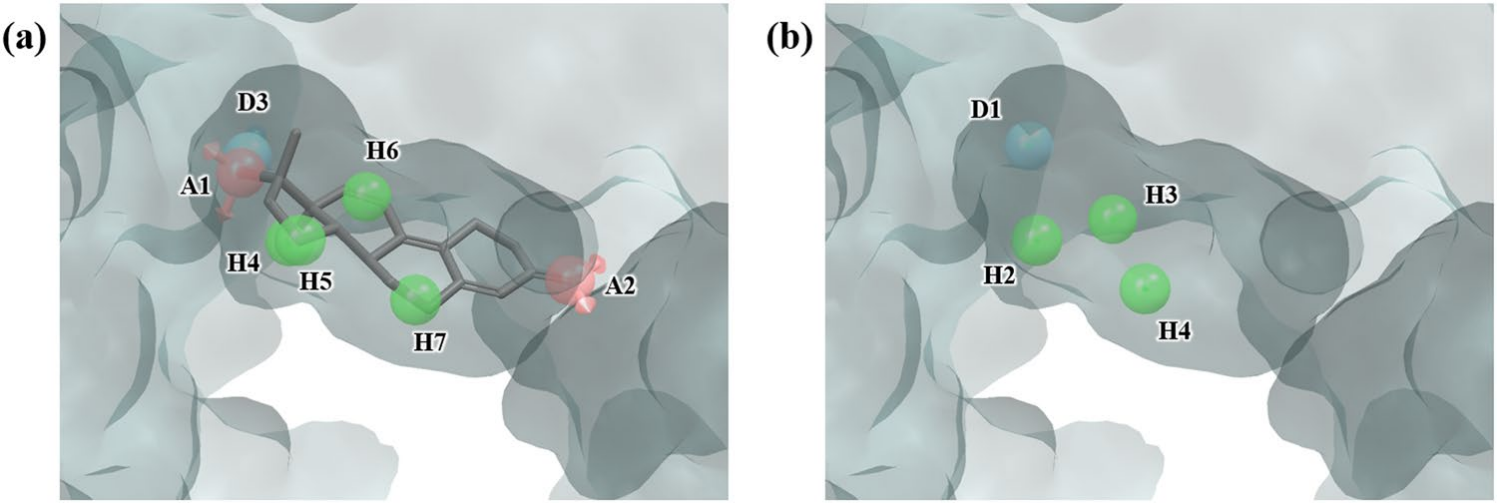
Ligand- and water-based pharmacophore model of AR. (**a**) 7 pharmacophore features of the ligand metribolone, AADHHHH, were generated by PHASE. (**b**) 4 pharmacophore features, DHHH, were generated by water pharmacophore model generation method. The pharmacophore features are numbered to help identification of each site and colored as follows: hydrogen bond acceptor (A), pink; hydrogen bond donor (D), skyblue; hydrophobic (H), green. To clarify the binding site of AR, the molecular surface is only shown.

AR is a nuclear receptor which consists of N-terminal domain, DNA-binding domain (DBD), and ligand-binding domain (LBD). If androgens, such as testosterone and 5α-dihydrotestosterone, bind to LDB, AR binds with DNA in the form of homodimer and regulate gene expression. AR is involved in a wide variety of physiological processes, especially control of male sexual differentiation. Since abnormality in AR function can lead to prostate cancer or benign prostatic hyperplasia (BHP), AR is an important drug target^30,31^. We used PDB structure 1XQ2, which was crystallized with the ligand metribolone. Metribolone produces 7 total pharmacophore features when run through PHASE (AADHHHH) (Fig. 4a). We identified 11 hydration sites inside the LBD of AR (Fig. S4 and Table S1). Our WP procedure yielded 4 features DHHH (Fig. 4b). The WP features overlap with the ligand pharmacophore features. Water-pharmacophore performed slightly better than docking in enrichment results at 1% and 5%, while docking performed slightly better than WP in enrichment results at 10% (Tables 2 and 3).

### Progesterone receptor (PR)

**Figure 5:**
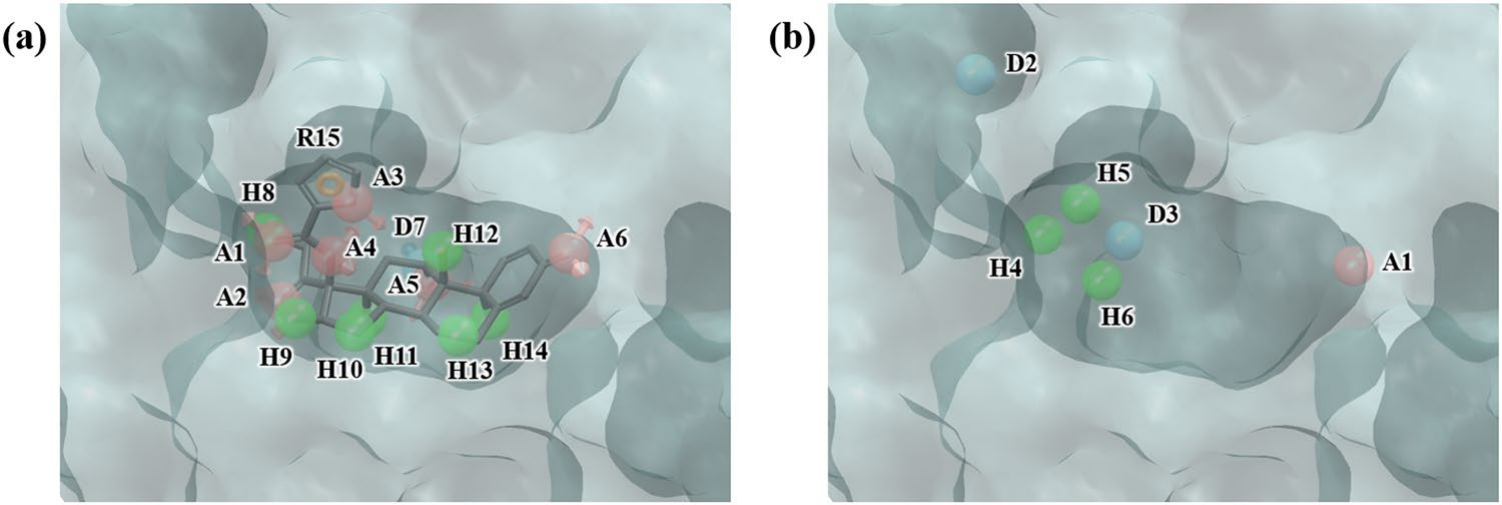
Ligand- and water-based pharmacophore model of PR. (**a**) 15 pharmacophore features of the ligand mometasone furoate, AAAAAADHHHHHHHR, were generated by PHASE. (**b**) 6 pharmacophore features, ADDHHH, were generated by water pharmacophore model generation method. The pharmacophore features are numbered to help identification of each site and colored as follows: hydrogen bond acceptor (A), pink; hydrogen bond donor (D), skyblue; hydrophobic (H), green; aromatic ring (R), orange. To clarify the binding site of PR, the molecular surface is only shown.

PR is another nuclear receptor which when combined with progesterone in cytosol would form a dimer and then regulate gene expression by binding DNA thereby controlling female pregnancy and menstruation. PR is a therapeutic target for miscarriage, uterine cancer, or breast cancer^32–34^. In 1SR7, the PDB structure used for our experiments, mometasone furoate, a potent steroid agonist, is bound in the ligand binding pocket of PR. PHASE produces a total of 15 ligand pharmacophore features (AAAAAADHHHHHHHR) for mometasone furoate. Hydration site analysis yielded 18 hydration sites (Fig. S5 and Table S2), some of which coincide with the ligand pharmacophore features (Fig. 5a). The WP generated 6 features, ADDHHH (Fig. 5b). Of these, except for the D2 and H5 features which is located on outside of binding site and on the ring group of the ligand (R15), all the other features overlap with the ligand pharmacophore features. Overall, screening with WP model resulted in better enrichment than screening by docking in enrichment results at 1%, 5%, and 10% (Tables 2 and 3).

### Retinoid X receptor alpha (RXRα)

**Figure 6:**
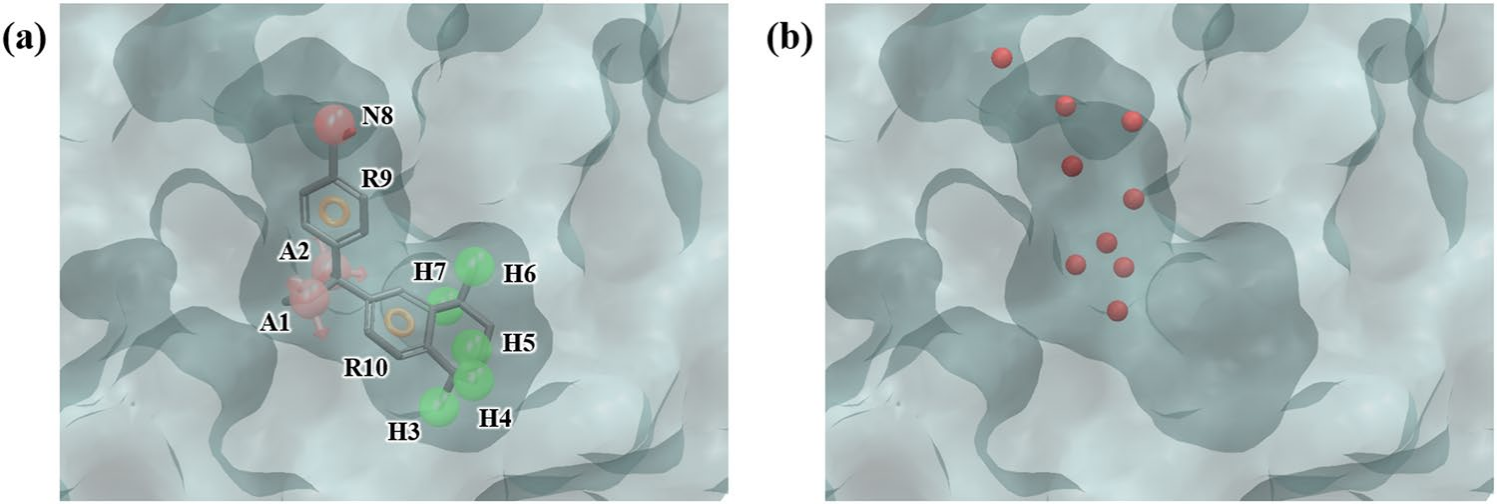
Ligand-based pharmacophore model and hydration sites of RXRα. (**a**) 10 pharmacophore features of the ligand BMS649, AAHHHHHNRR, were generated by PHASE. (**b**) 9 hydration sites generated by MD simulations and hydration site analysis were displayed as red spheres. The pharmacophore features are numbered to help identification of each site and colored as follows: hydrogen bond acceptor (A), pink; hydrophobic (H), green; negative (N), red; aromatic ring (R), orange. To clarify the binding site of RXRα, the molecular surface is only shown.

RXRα is a nuclear receptor and plays a role as a transcription factor controlling various physiological processes such as cell development, apoptosis, and homeostasis. The natural ligand for RXRα is 9-cis-retinoic acid (9-cis-RA) and together with synthetic ligands, it is known to be effective in inhibition of tumor formation rendering RXRα to be an anticancer drug target^35,36^. In the PDB structure 1MVC, which is used for our test, a synthetic agonist compound BMS649 is bound to the ligand binding domain (LBD). BMS649 produces 10 pharmacophore features when run through PHASE, AAHHHHHNRR (Fig. 6a). Hydration site analysis yielded 9 hydration sites (Fig. S6 and Table S3), but they were all near the opening of the binding site, rather than the LBD (Fig. 6b). This is due to the fact that the binding site is hydrophobic and thus the water molecules cannot penetrate deep inside the pocket prohibiting proper water sampling in the LBD (Fig. S11). By the criteria for WP, no feature was generated, and thus no compound was screened. This case confirms that our method of WP does not suit well the cases in which binding pockets are strongly hydrophobic and water is not sampled thoroughly. On the other hand, docking showed better enrichment results at 1%, 5%, and 10% (Tables 2 and 3).

### Glucocorticoid receptor (GR)

**Figure 7:**
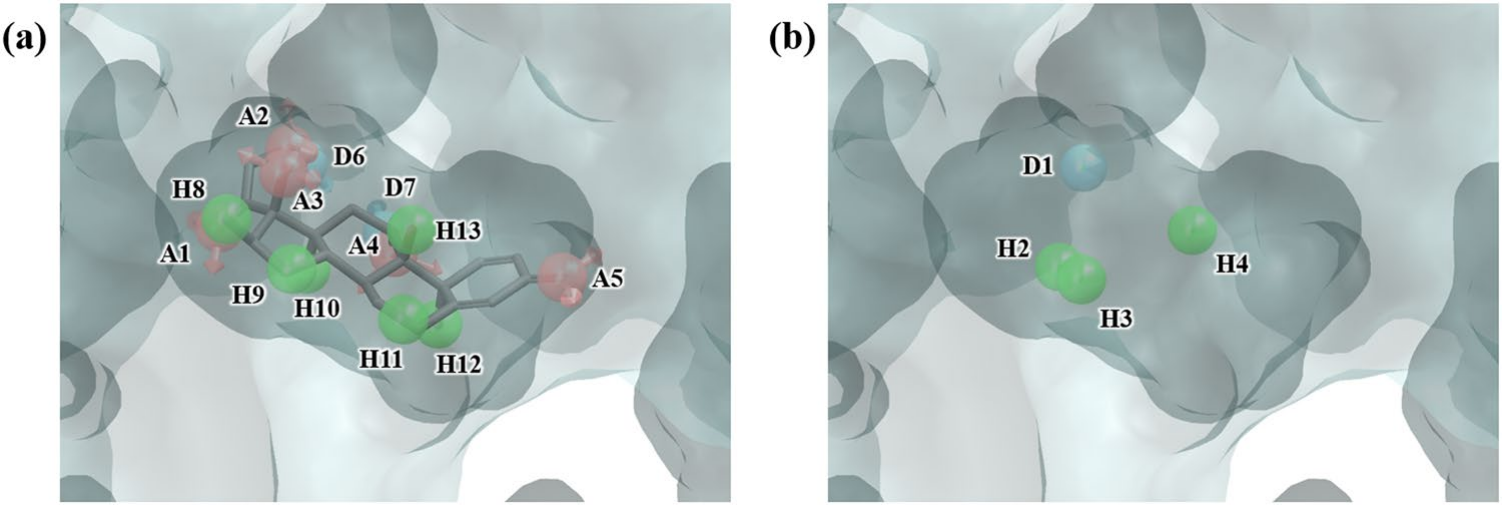
Ligand- and water-based pharmacophore model of GR. (**a**) 13 pharmacophore features of the ligand dexamethasone, AAAAADDHHHHHH, were generated by PHASE. (**b**) 4 pharmacophore features, DHHH, were generated by water pharmacophore model generation method. The pharmacophore features are numbered to help identification of each site and colored as follows: hydrogen bond acceptor (A), pink; hydrogen bond donor (D), skyblue; hydrophobic (H), green. To clarify the binding site of GR, the molecular surface is only shown.

GR is a nuclear receptor which binds DNA when combined with steroid hormones and acts as a transcription factor. It is involved in glucose homeostasis, bone turnover, cell differentiation, lung maturation, and inflammation. Abnormality in GR can cause Cushing’s syndrome, autoimmune diseases, and various kinds of cancer and thus has been studied as an important therapeutic target^37–39^. The structure used in our experiment is 1M2Z, in which dexamethasone is bound in the ligand binding pocket of GR. PHASE produces 13 pharmacophore features (AAAAADDHHHHHH) (Fig. 7a). Hydration site analysis produced 19 hydration sites (Fig. S7 and Table S4), from which a 4-featured WP model, DHHH, was generated (Fig. 7b). In an enrichment study, the WP model screening yielded significantly better result than screening by docking in enrichment results at 1%, 5%, and 10% (Tables 2 and 3).

### Peroxisome proliferator-activated receptor gamma (PPARγ)

**Figure 8:**
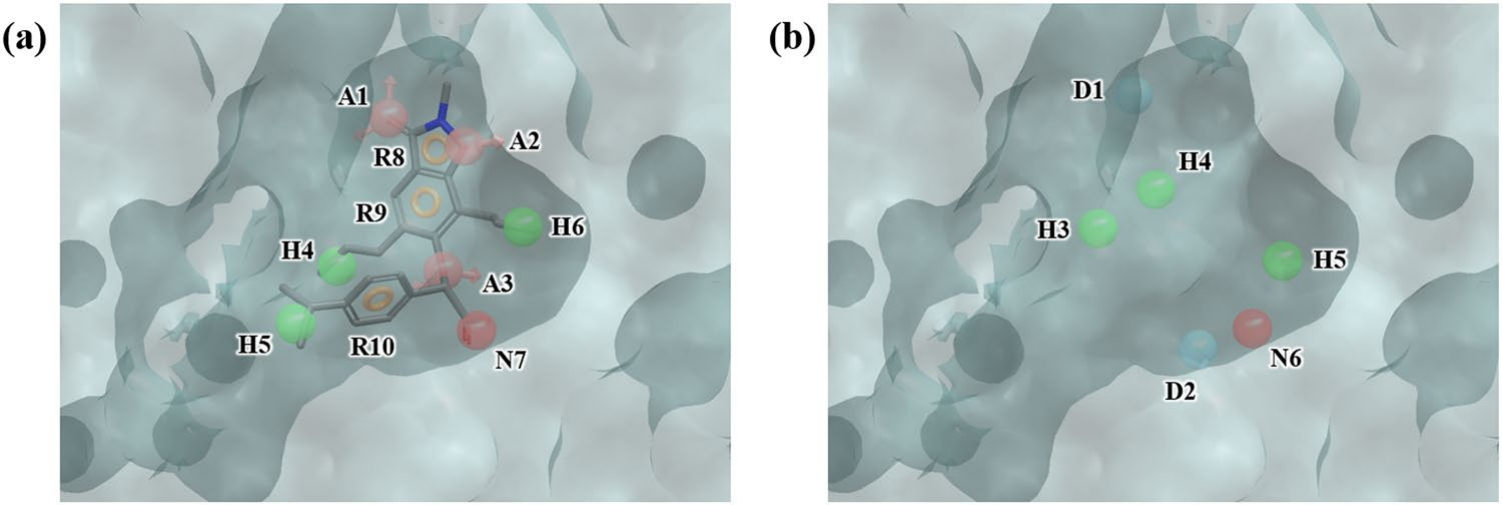
Ligand- and water-based pharmacophore model of PPARγ. (**a**) 10 pharmacophore features of the ligand α-aryloxyphenylacetic acid, AAAHHHNRRR, were generated by PHASE. (**b**) 6 pharmacophore features, DDHHHN, were generated by water pharmacophore model generation method. The pharmacophore features are numbered to help identification of each site and colored as follows: hydrogen bond acceptor (A), pink; hydrogen bond donor (D), skyblue; hydrophobic (H), green; negative (N), red. To clarify the binding site of PPARγ, the molecular surface is only shown.

PPARγ is a nuclear receptor, which controls fatty acid storage and glucose metabolism. It is related to various diseases, such as obesity, diabetes, atherosclerosis, and cancer^40–42^. The structure used in our experiment 1FM9, is bound with α-aryloxyphenylacetic acid, which is a PPARγ agonist. PHASE produced 10 pharmacophore features (AAAHHHNRRR) (Fig. 8a). Hydration site analysis yielded 25 hydration sites (Fig. S8 and Table S5) and from these 6 WP features were selected, DDHHHN (Fig. 8b). Although WP screening selected a few actives, enrichment factors for WP were minimal, with positive number only for 1% case. In comparison, docking performed well with enrichment factors of more than 10 across 1%, 5%, and 10% (Tables 2 and 3). This case reveals the limitation of WP method. With large binding sites, pharmacophore models, for their imprecision on geometry of the ligands, often fail to filter out ligands that may not have correct interactions with the binding site.

### Poly (ADP-ribose) polymerase (PARP)

**Figure 9:**
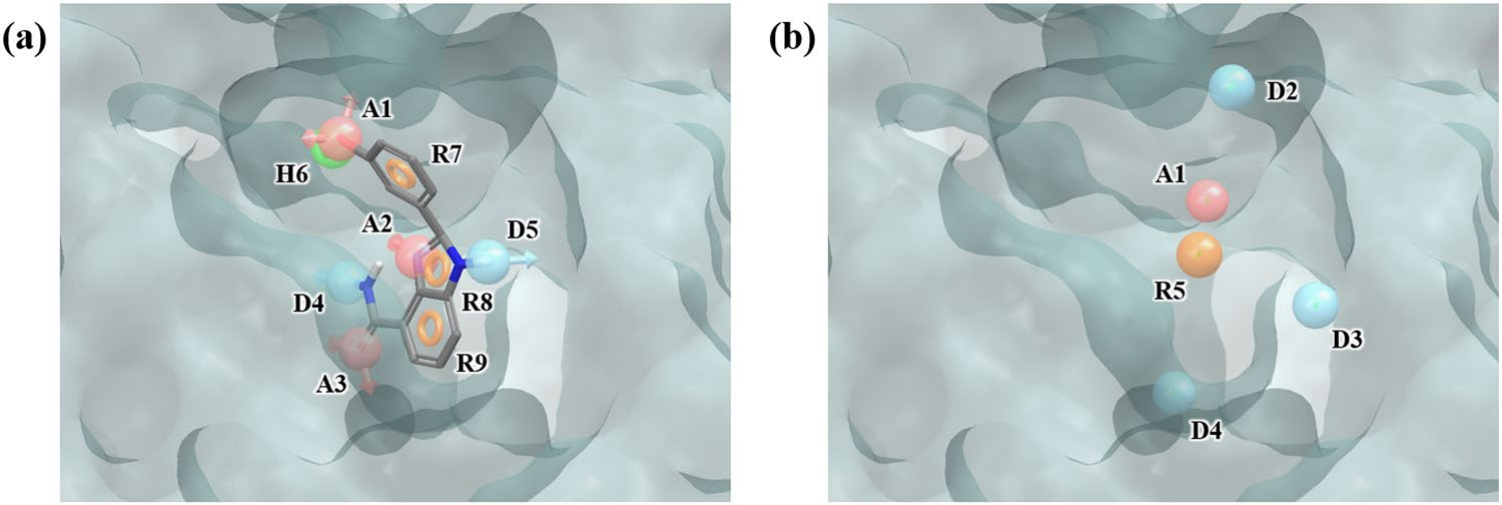
Ligand- and water-based pharmacophore model of PARP. (**a**) 9 pharmacophore features of the ligand 2-(3-methoxyphenyl)-1H-benzimidazole-4-carboxamide, AAADDHRRR, were generated by PHASE. (**b**) 5 pharmacophore features, ADDDR, were generated by water pharmacophore model generation method. The pharmacophore features are numbered to help identification of each site and colored as follows: hydrogen bond acceptor (A), pink; hydrogen bond donor (D), skyblue; hydrophobic (H), green; aromatic ring (R), orange. To clarify the binding site of PARP, the molecular surface is only shown.

PARP is a nuclear enzyme, which plays an important role in DNA repair and is related to cancer. It was found that inhibition of PARP in tumor cells can strengthen the effect of radiotherapy and DNA-targeted chemotherapy^43^. Our HSA yielded 36 hydration sites, which was considerably more than other targets. PHASE produced 9 pharmacophore features on the ligand 2-(3-methoxyphenyl)-1*H*-benzimidazole-4-carboxamide (AAADDHRRR) (Fig. 9a) and the WP model 5 features of ADDDR (Fig. 9b) was generated from 36 hydration sites (Fig. S9 and Table S6). In enrichment studies, docking failed to select active ligands at all level (Tables 2 and 3). This failure may be explained by observing that the binding site is significantly larger than a usual “fragment-like” ligand and therefore differentiating the actual actives from decoys becomes more difficult as there are much more possibilities of fitting ligands in the binding site. On the other hand, WP performed rather well, achieving enrichment factor of 22.6 at 1%, 5.2 at 5%, and 2.6 at 10%.

### Acetylcholinesterase (AChE)

**Figure 10:**
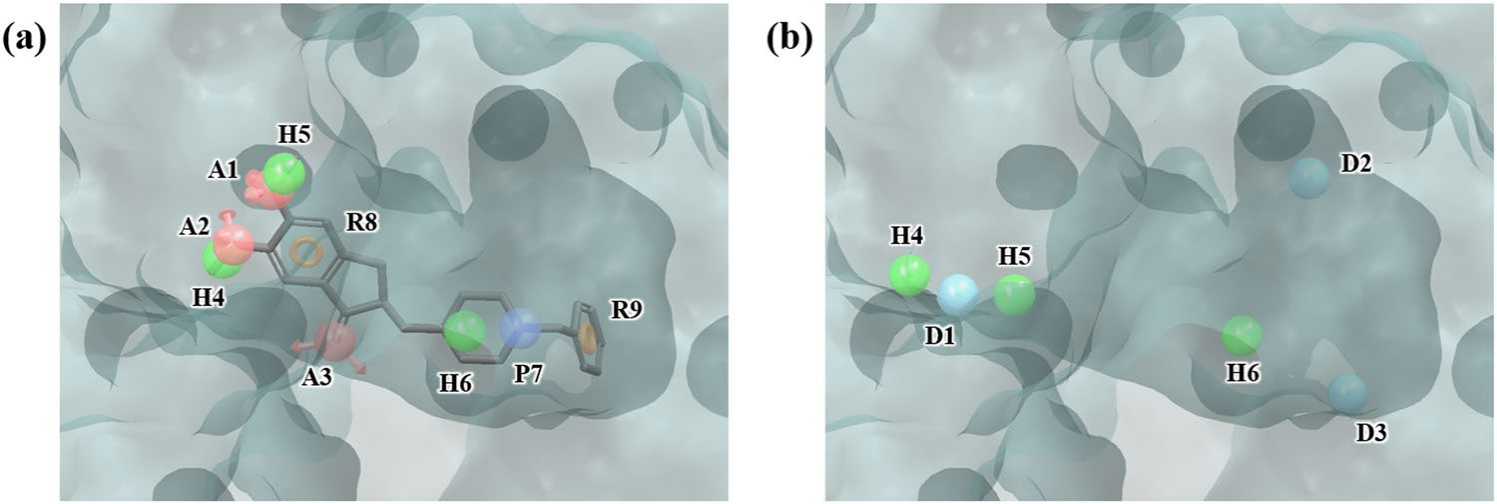
Ligand- and water-based pharmacophore model of AChE. (**a**) 9 pharmacophore features of the ligand donepezil, AAAHHHPRR, were generated by PHASE. (**b**) 6 pharmacophore features, DDDHHH, were generated by water pharmacophore model generation method. The pharmacophore features are numbered to help identification of each site and colored as follows: hydrogen bond acceptor (A), pink; hydrogen bond donor (D), skyblue; hydrophobic (H), green; positive (P), blue; aromatic ring (R), orange. To clarify the binding site of AChE, the molecular surface is only shown.

AChE is an enzyme that hydrolyzes a neurotransmitter acetylcholine. It is involved in synaptic transmission and thus is an important therapeutic target for neurodegenerative diseases such as Alzheimer’s disease^44^. The structure 4EY7, used in our experiment, is bound with donepezil, which is known to be effective for neurodegenerative diseases. PHASE produced 9 ligand pharmacophore features for donepezil, AAAHHHPRR (Fig. 10a). HSA produced 43 hydration sites (Fig. S10 and Table S7) and the WP algorithm produced 6 features of DDDHHH (Fig. 10b). In this case, WP did not perform as well as docking at all level (Tables 2 and 3). This poor performance by WP is probably due to the fact that the binding site is large and narrow, which makes pharmacophore model-based filtering process vulnerable to exact geometry of the compound structures. While WP can produce compounds that have relevant interactions with the receptor, these compounds do not necessarily have the right dimensions as pharmacophore filtering do not consider geometries of the ligands being filtered.

Our WP method successfully generated the pharmacophore models derived from the water molecules without known ligands for all 7 targets. When compared with the pharmacophore models derived from known ligands, our pharmacophore models had similar features which are essential for binding target proteins. This shows that even without any knowledge of active ligands, WP can generate a pharmacophore model solely from the binding site structure. An enrichment study was conducted to validate the performance of the method as compared with Glide docking which has been proven to perform well on a wide variety of targets. The results showed that WP outperformed Glide docking in 4 targets out of 7 in enrichment factor at 1%. Time-wise, although WP requires MD simulations beforehand, pharmacophore filtering takes much less than docking. The limitations of WP were also observed when the binding sites were too hydrophobic like RXRα case. Hydrophobic regions in protein binding sites are almost always hydrated and hence WP should be able to capture those points. However, in the case of RXRα, the entrance to the binding site is very narrow and therefore water molecules do not freely venture into the binding site during MD simulations. This fact results in an inadequate sampling of the hydration points, which in turn gives a pharmacophore model that may be non-druglike leading to poor enrichment performance. WP also had problem with large pockets that can accommodate large sized ligands such as PPARγ and AChE (Fig. S11). In these cases, we believe that WP can be combined with docking in order to improve its efficacy. Further work is under way to heighten the usefulness of WP method along this line.

## Conclusions

In this paper, we demonstrated the feasibility of generating pharmacophore models based purely on the receptor structure through probing the protein binding-site surface with explicit water molecular dynamics simulations. We have introduced a method to construct the water-based pharmacophore and demonstrated that such pharmacophore is able to explore chemical space that is explored using more traditional ligand-based approaches. We have further demonstrated that water pharmacophore can be used for virtual ligand screening processes with a performance that compares well with established docking methods shown by enrichment studies. Our WP method selects feature sites based on thermodynamic criteria, therefore selected sites are more likely to be meaningful interaction features than random selection. This technique can be used as a standalone approach such as pharmacophore-based virtual screening when known binder data is lacking. The technique also can be incorporated into the ligand-based approaches. The more important molecular features derived from the known binder data can be selected by the consideration of WP information. In addition, structure-based approaches such as docking can be combined with WP. The two methods can then be applied sequentially in virtual screening. We envision that introducing localized solvation thermodynamics through Grid Inhomogeneous Solvation Theory^45^ or hydration site approaches such as WaterMap^46^ and STOW^47^ could help assign weights to individual pharmacophore sites and help improve searching and scoring schemes.

## Electronic supplementary material

Supplementary Information
