# Supplementary material for: Water Pharmacophore: Designing Ligands using Molecular Dynamics Simulations with Water
Source: Sci Rep. 2018 Jul 10;8:10400. doi: 10.1038/s41598-018-28546-z (PMC6039478; doi:10.1038/s41598-018-28546-z)
Supplement: Supplementary file 1 — Supplementary Information [file 41598_2018_28546_MOESM1_ESM.docx]

**SUPPLEMENTARY INFORMATION**

**Water Pharmacophore: Designing Ligands using Molecular Dynamics Simulations with Water**

Sang Won Jung^1,3^, Minsup Kim^1^, Steven Ramsey^2^, Tom Kurtzman^2,*^, and Art E. Cho^1,*^

^1^ *Department of Bioinformatics, Korea University, Sejong, 30019, Korea*

^2^ *Department of Chemistry, Lehman College, Bronx, New York 10468, USA*

^3^ *Present address: Center for Supercomputing and Big Data, DGIST, Daegu 42988, Korea*

* Corresponding Authors : [simpleliquid@gmail.com](mailto:simpleliquid@gmail.com) and [artcho@korea.ac.kr](mailto:artcho@korea.ac.kr)


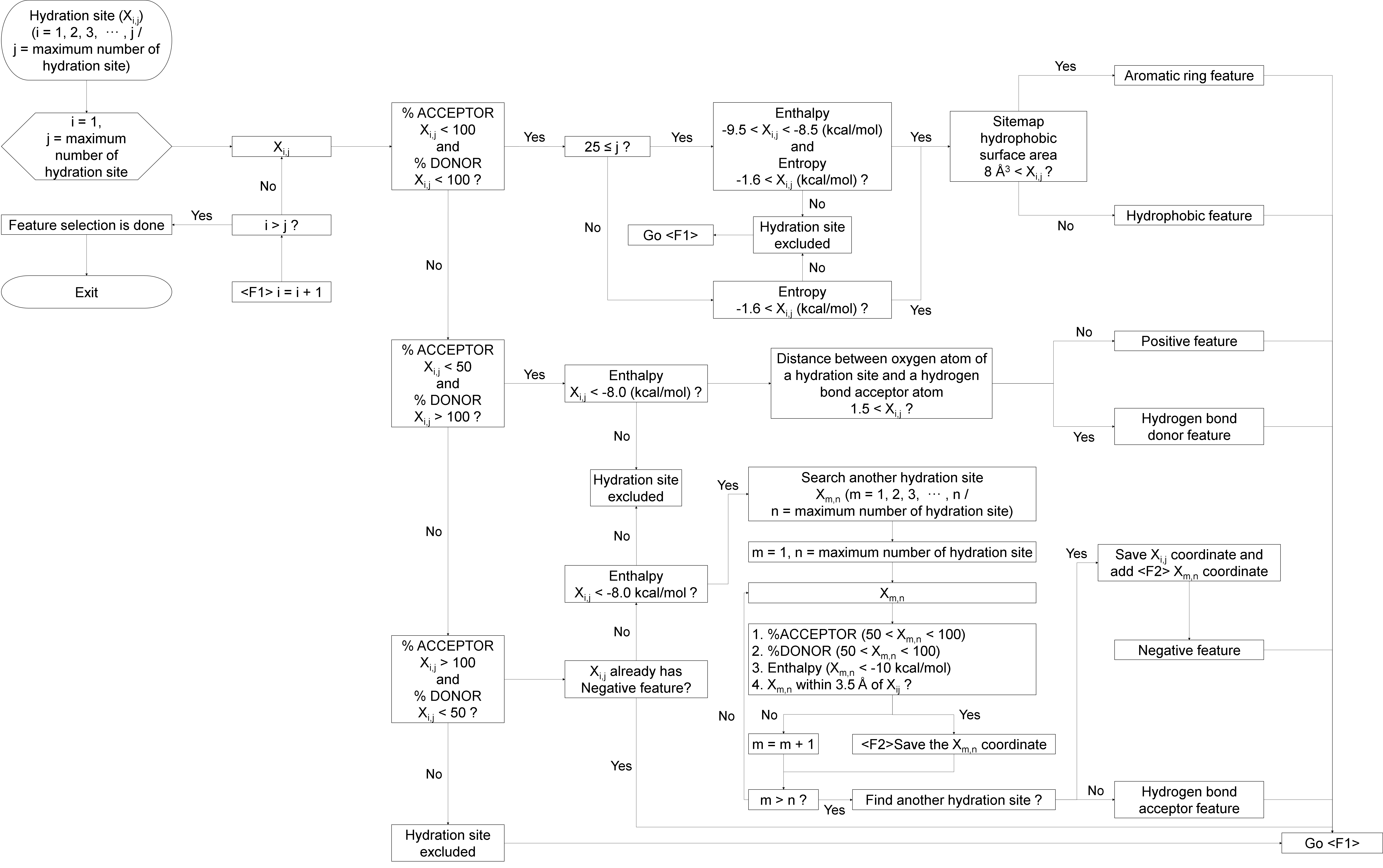


**Figure 1.** Scheme documenting the process of assigning pharmacophore features


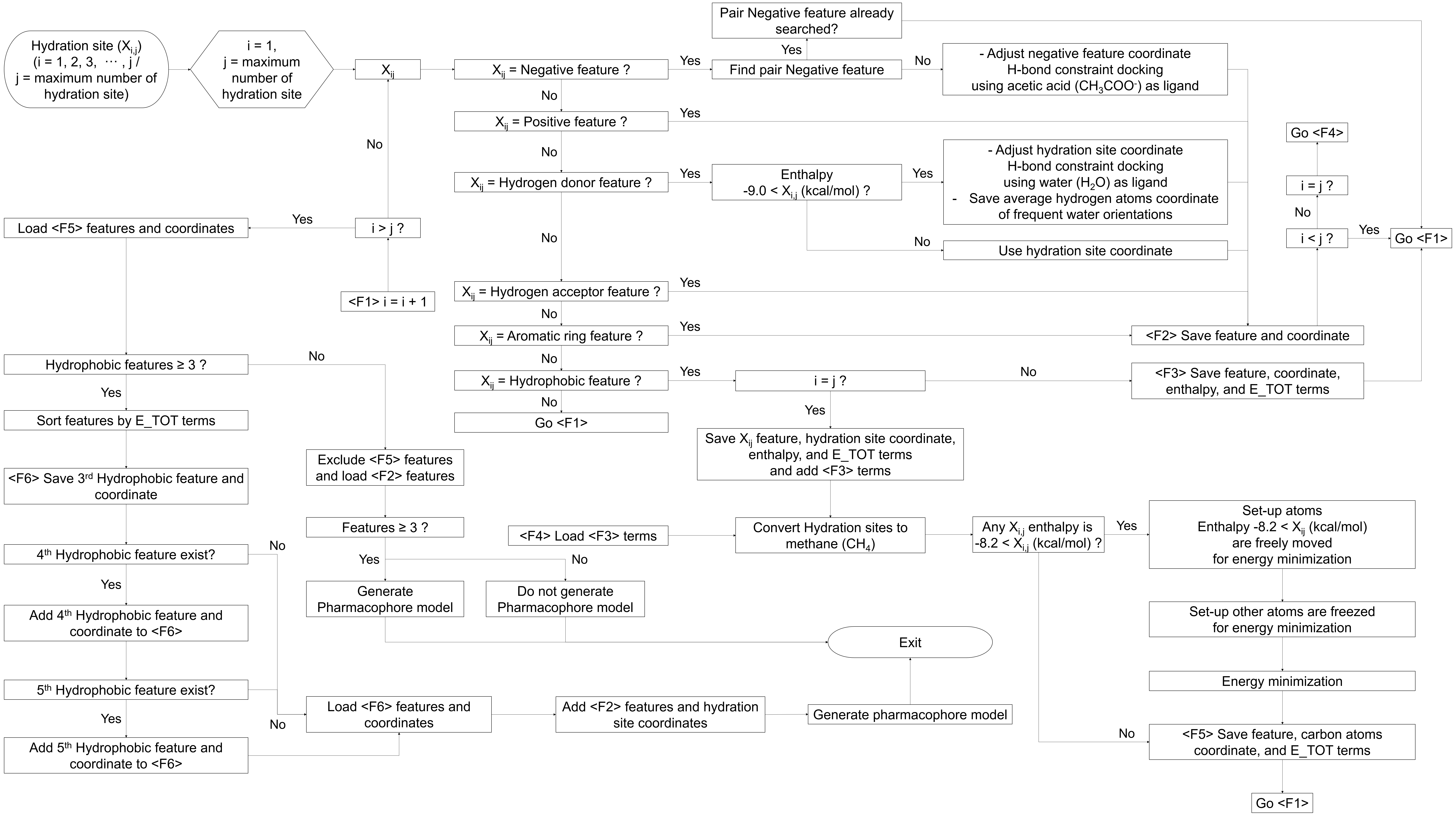


**Figure 2.** Scheme documenting the process of determining water pharmacophore model


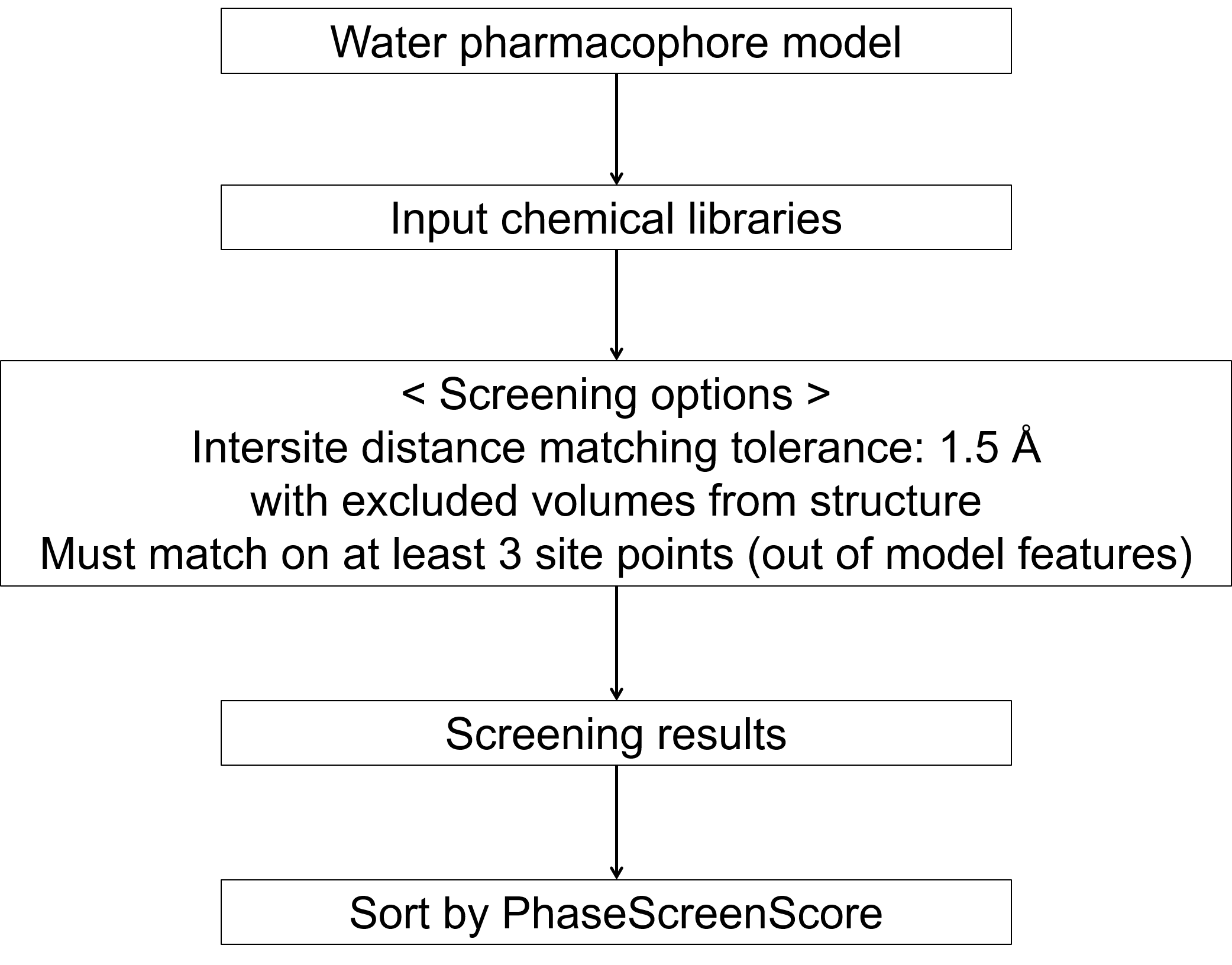


**Figure 3.** Scheme documenting the process of screening against the water pharmacophore model


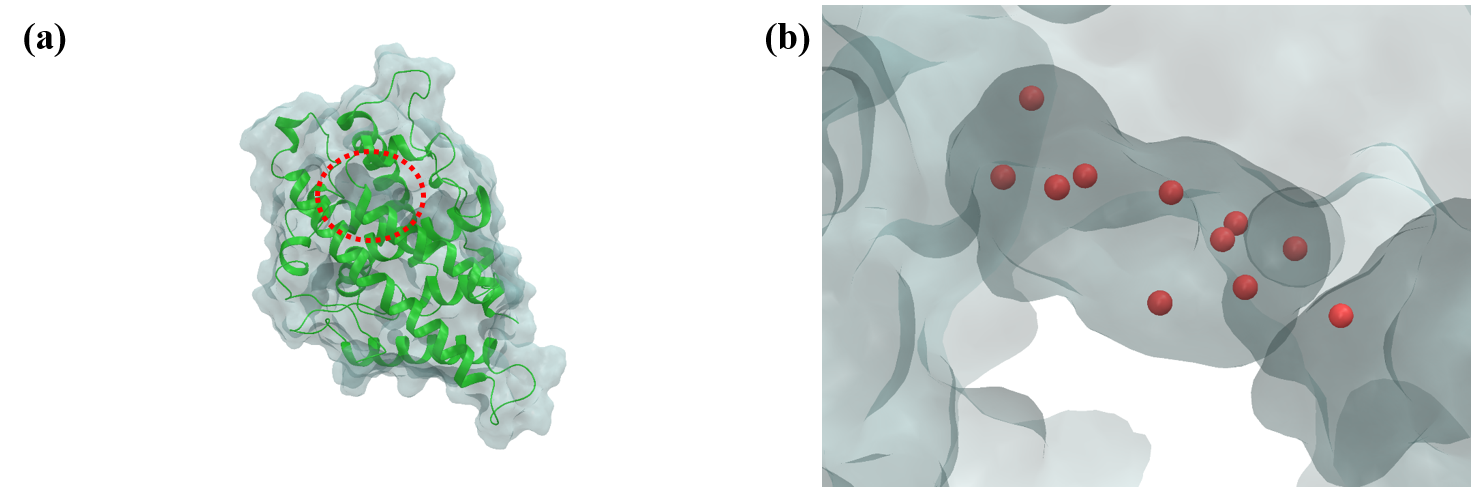


**Figure 4.** Binding site and hydration sites of AR. (a) Ribbon and surface representations of AR. Binding site is indicated by red dotted circle. (b) 11 hydration sites (red spheres) were found in the binding site of AR.


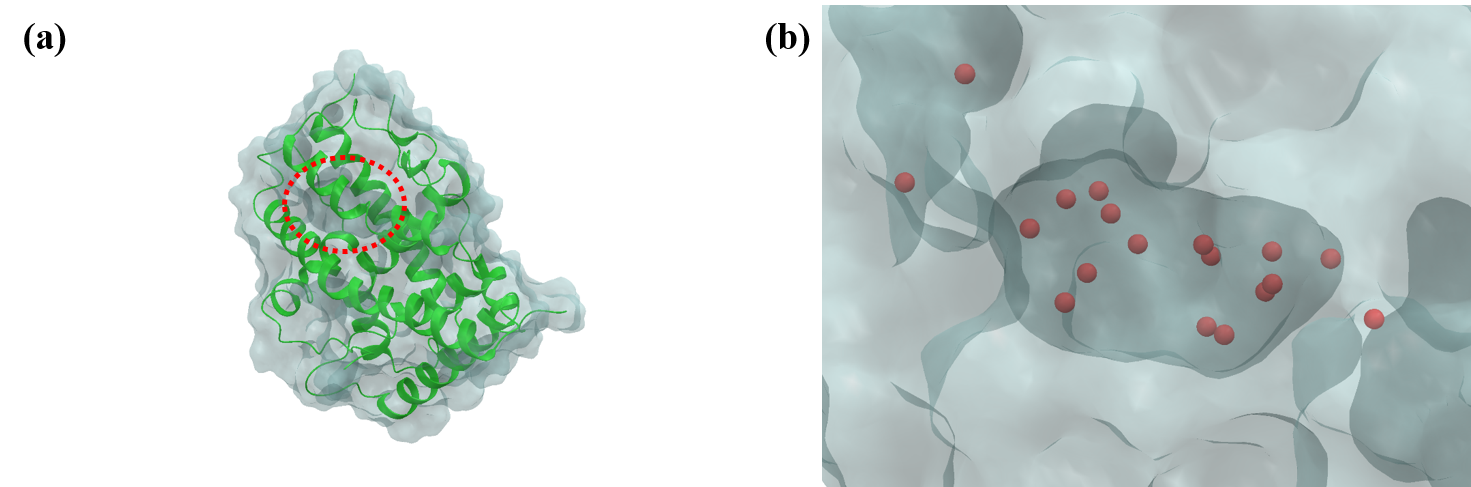


**Figure 5.** Binding site and hydration sites of PR. (a) Ribbon and surface representations of PR. Binding site is indicated by red dotted circle. (b) 18 hydration sites (red spheres) were found in the binding site of PR.


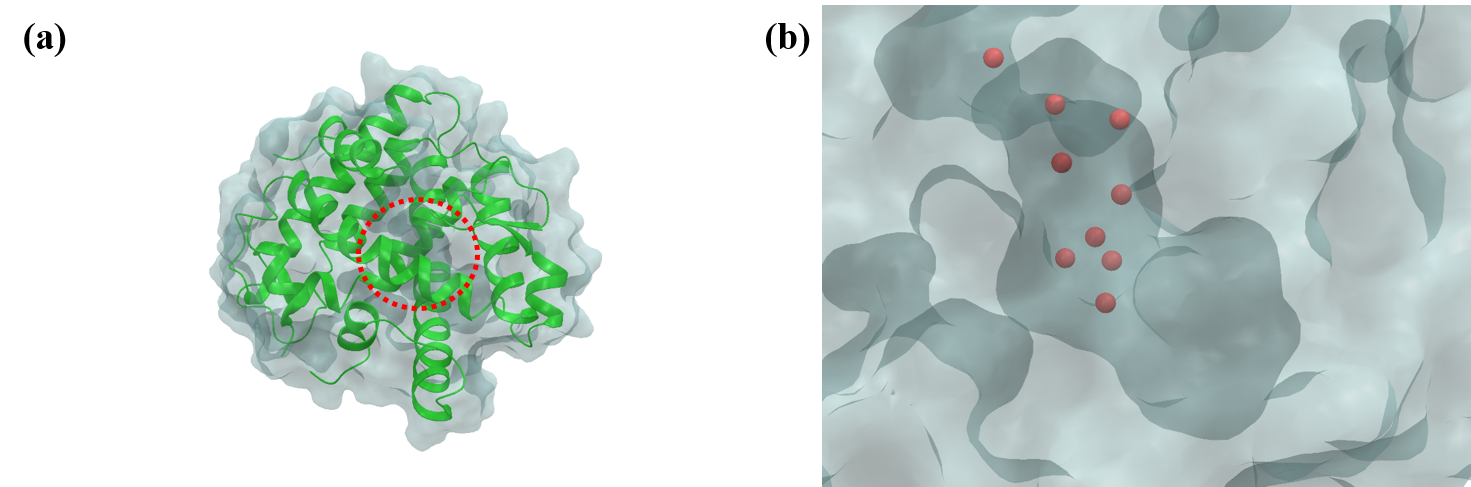


**Figure 6.** Binding site and hydration sites of RXRα. (a) Ribbon and surface representations of RXRα. Binding site is indicated by red dotted circle. (b) 9 hydration sites (red spheres) were found in the binding site of RXRα.


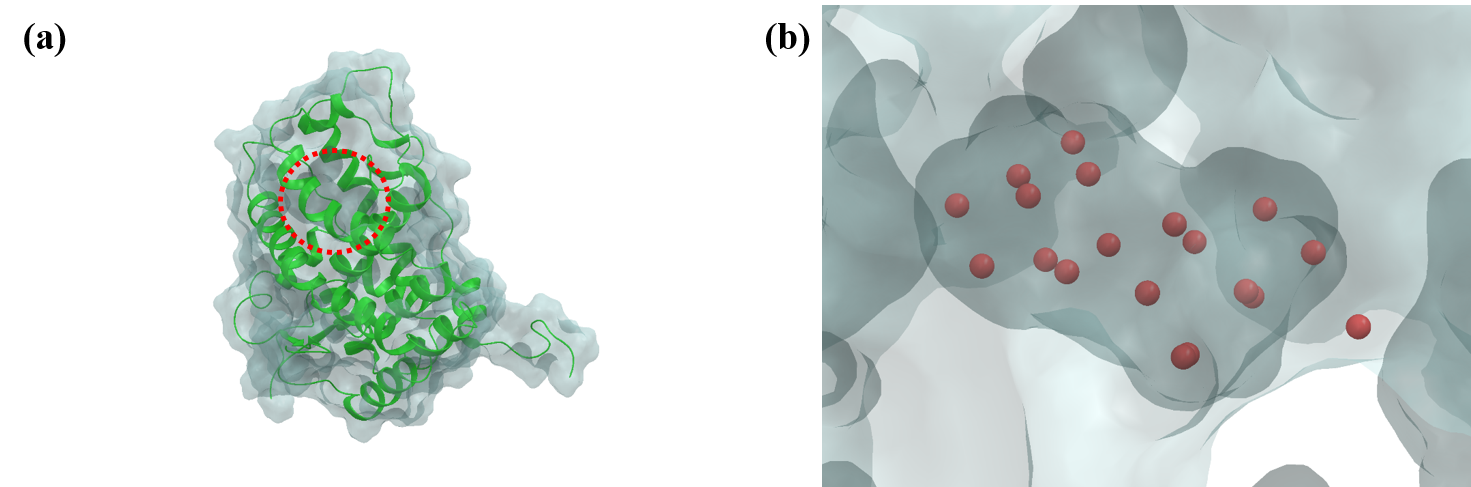


**Figure 7.** Binding site and hydration sites of GR. (a) Ribbon and surface representations of GR. Binding site is indicated by red dotted circle. (b) 19 hydration sites (red spheres) were found in the binding site of GR.


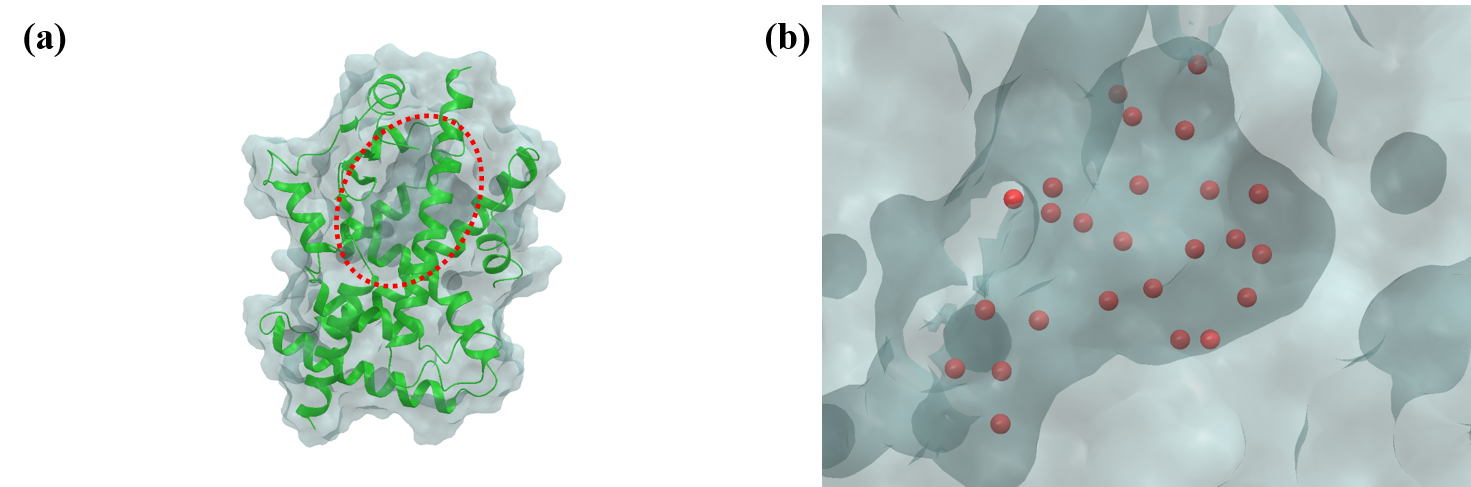


**Figure 8.** Binding site and hydration sites of PPARγ. (a) Ribbon and surface representations of PPARγ. Binding site is indicated by red dotted circle. (b) 25 hydration sites (red spheres) were found in the binding site of PPARγ.


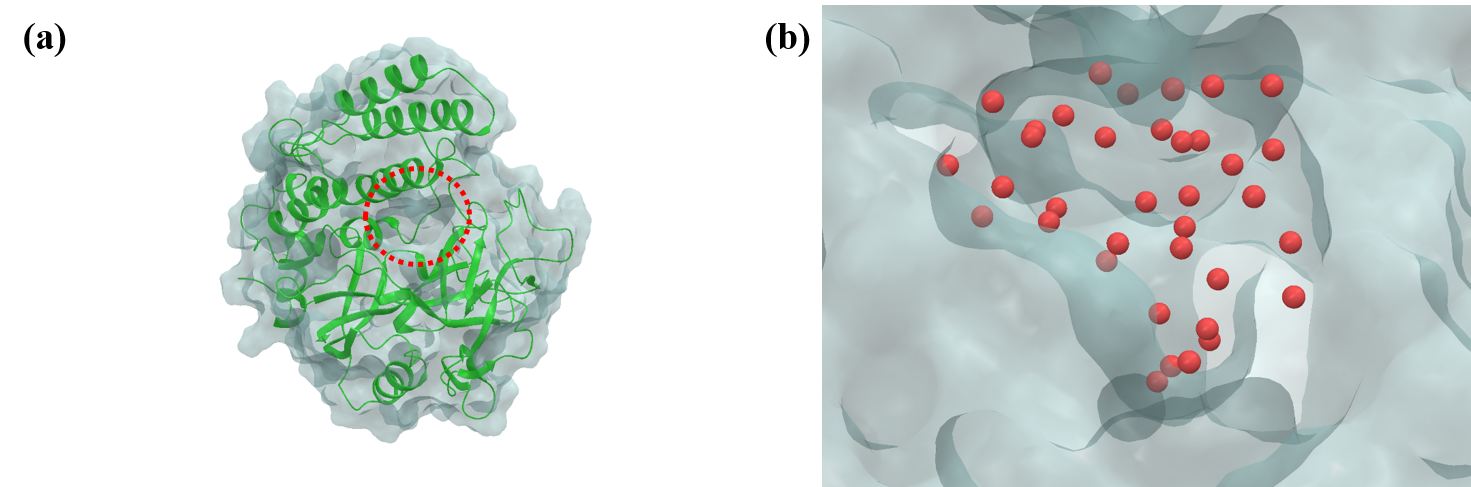


**Figure 9.** Binding site and hydration sites of PARP. (a) Ribbon and surface representations of PARP. Binding site is indicated by red dotted circle. (b) 36 hydration sites (red spheres) were found in the binding site of PARP.


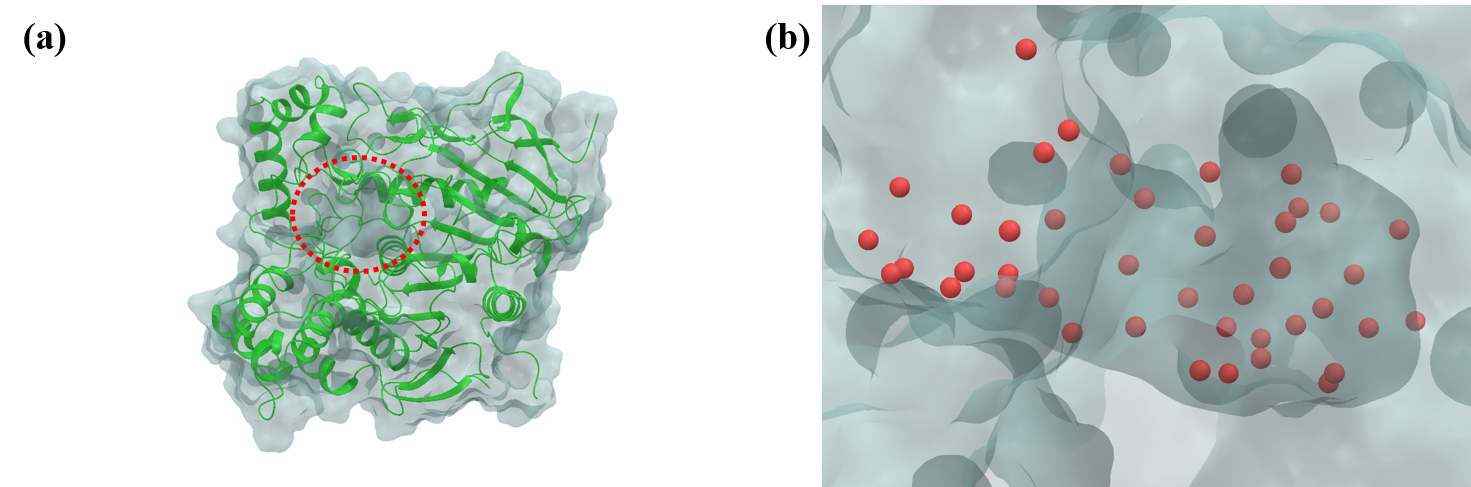


**Figure 10.** Binding site and hydration sites of AChE. (a) Ribbon and surface representations of AChE. Binding site is indicated by red dotted circle. (b) 43 hydration sites (red spheres) were found in the binding site of AChE.


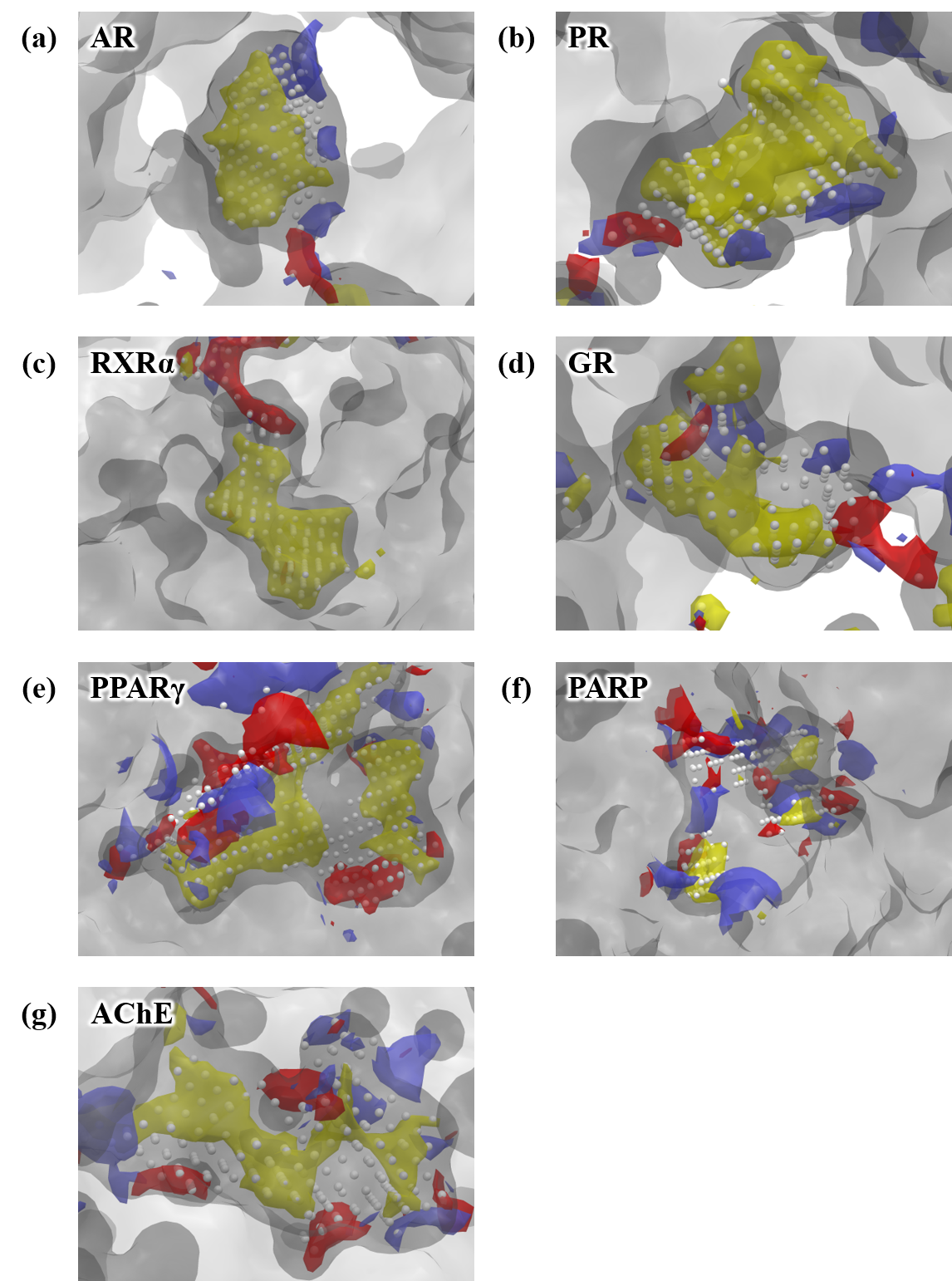


**Figure 11.** Binding site analysis of the seven targets using SiteMap. *Yellow* regions correspond to favorable hydrophobic interactions. *Red* and *Blue* regions correspond to favorable hydrogen bond acceptor and donor interactions, respectively.

**Table 1.** Hydration sites analysis of AR. Each hydration sites were numbered from the highest to lowest count of oxygen atom of water. The enthalpic and entropic energies of each hydration sites and the sum of both energies, E_TOT, were presented. The ratios of hydrogen bonding donors and acceptors are also presented. In addition, pharmacophore feature assigned by our scheme is presented and the selected feature for the model is highlighted by yellow shading.

| **Number** | **Enthalpy**  **(kcal/mol)** | **Entropy**  **(kcal/mol)** | **E_TOT**  **(kcal/mol)** | **%DONOR** | **%ACCEPTOR** | **Pharmacophore feature** |
| --- | --- | --- | --- | --- | --- | --- |
| 1 | -10.548 | -3.351 | -13.900 | 31 | 114 | - |
| 2 | -8.947 | -3.374 | -12.322 | 141 | 0 | D |
| 3 | -10.386 | -3.146 | -13.531 | 45 | 22 | - |
| 4 | -9.062 | -2.381 | -11.443 | 84 | 0 | - |
| 5 | -9.109 | -2.117 | -11.226 | 4 | 76 | - |
| 6 | -8.542 | -1.976 | -10.518 | 80 | 0 | - |
| 7 | -8.054 | -1.387 | -9.442 | 83 | 0 | H |
| 8 | -8.145 | -1.406 | -9.550 | 17 | 0 | H |
| 9 | -8.554 | -1.545 | -10.100 | 7 | 0 | H |
| 10 | -7.261 | -1.165 | -8.426 | 31 | 0 | H |
| 11 | -7.892 | -1.471 | -9.363 | 32 | 0 | H |

**Table 2.** Hydration sites analysis of PR. Each hydration sites were numbered from the highest to lowest count of oxygen atom of water. The enthalpic and entropic energies of each hydration sites and the sum of both energies, E_TOT, were presented. The ratios of hydrogen bonding donors and acceptors are also presented. In addition, pharmacophore feature assigned by our scheme is presented and the selected feature for the model is highlighted by yellow shading.

| **Number** | **Enthalpy**  **(kcal/mol)** | **Entropy**  **(kcal/mol)** | **E_TOT**  **(kcal/mol)** | **%DONOR** | **%ACCEPTOR** | **Pharmacophore feature** |
| --- | --- | --- | --- | --- | --- | --- |
| 1 | -9.175 | -3.641 | -12.817 | 160 | 112 | - |
| 2 | -9.071 | -2.880 | -11.951 | 13 | 110 | A |
| 3 | -8.784 | -1.975 | -10.759 | 1 | 0 | - |
| 4 | -9.402 | -2.305 | -11.706 | 97 | 0 | - |
| 5 | -9.386 | -2.391 | -11.777 | 88 | 0 | - |
| 6 | -9.080 | -1.695 | -10.775 | 50 | 0 | - |
| 7 | -9.622 | -2.048 | -11.670 | 96 | 0 | - |
| 8 | -8.906 | -2.337 | -11.242 | 139 | 0 | D |
| 9 | -9.154 | -1.447 | -10.601 | 75 | 0 | H |
| 10 | -8.908 | -1.534 | -10.442 | 80 | 0 | H |
| 11 | -9.138 | -1.146 | -10.284 | 43 | 6 | H |
| 12 | -8.863 | -0.952 | -9.816 | 0 | 0 | H |
| 13 | -9.053 | -1.278 | -10.331 | 75 | 1 | H |
| 14 | -9.087 | -1.601 | -10.688 | 32 | 0 | - |
| 15 | -9.083 | -1.024 | -10.107 | 50 | 13 | H |
| 16 | -8.933 | -0.841 | -9.774 | 0 | 0 | H |
| 17 | -9.092 | -0.746 | -9.839 | 8 | 0 | H |
| 18 | -9.441 | -1.837 | -11.277 | 153 | 0 | D |

**Table 3.** Hydration sites analysis of RXRα. Each hydration sites were numbered from the highest to lowest count of oxygen atom of water. The enthalpic and entropic energies of each hydration sites and the sum of both energies, E_TOT, were presented. The ratios of hydrogen bonding donors and acceptors are also presented. In addition, pharmacophore feature assigned by our scheme is presented and the selected feature for the model is highlighted by yellow shading.

| **Number** | **Enthalpy**  **(kcal/mol)** | **Entropy**  **(kcal/mol)** | **E_TOT**  **(kcal/mol)** | **%DONOR** | **%ACCEPTOR** | **Pharmacophore feature** |
| --- | --- | --- | --- | --- | --- | --- |
| 1 | -11.672 | -4.299 | -15.971 | 31 | 153 | N |
| 2 | -11.825 | -4.934 | -16.759 | 93 | 84 | N |
| 3 | -9.326 | -3.984 | -13.310 | 75 | 0 | - |
| 4 | -7.967 | -4.050 | -12.017 | 87 | 0 | - |
| 5 | -7.627 | -2.703 | -10.330 | 76 | 3 | - |
| 6 | -9.516 | -3.289 | -12.805 | 42 | 109 | A |
| 7 | -9.051 | -2.876 | -11.927 | 0 | 0 | - |
| 8 | -8.933 | -1.420 | -10.353 | 8 | 0 | H |
| 9 | -8.785 | -1.358 | -10.143 | 14 | 0 | H |

**Table 4.** Hydration sites analysis of GR. Each hydration sites were numbered from the highest to lowest count of oxygen atom of water. The enthalpic and entropic energies of each hydration sites and the sum of both energies, E_TOT, were presented. The ratios of hydrogen bonding donors and acceptors are also presented. In addition, pharmacophore feature assigned by our scheme is presented and the selected feature for the model is highlighted by yellow shading.

| **Number** | **Enthalpy**  **(kcal/mol)** | **Entropy**  **(kcal/mol)** | **E_TOT**  **(kcal/mol)** | **%DONOR** | **%ACCEPTOR** | **Pharmacophore feature** |
| --- | --- | --- | --- | --- | --- | --- |
| 1 | -10.719 | -3.351 | -14.069 | 121 | 132 | - |
| 2 | -9.020 | -2.721 | -11.740 | 68 | 0 | - |
| 3 | -9.400 | -2.760 | -12.160 | 89 | 0 | - |
| 4 | -9.558 | -2.702 | -12.260 | 11 | 80 | - |
| 5 | -9.490 | -2.280 | -10.769 | 0 | 85 | - |
| 6 | -10.007 | -2.284 | -12.291 | 85 | 0 | - |
| 7 | -8.703 | -1.939 | -10.642 | 11 | 0 | - |
| 8 | -8.654 | -2.162 | -10.816 | 54 | 1 | - |
| 9 | -8.444 | -1.415 | -9.859 | 1 | 0 | H |
| 10 | -9.583- | -2.213 | -11.796 | 84 | 0 | - |
| 11 | -8.004 | -2.232 | -10.236 | 128 | 0 | D |
| 12 | -9.364 | -1.847 | -11.211 | 88 | 0 | - |
| 13 | -7.757 | -1.622 | -9.379 | 58 | 0 | - |
| 14 | -8.715 | -0.768 | -9.482 | 3 | 0 | H |
| 15 | -8.410 | -1.100 | -9.510 | 25 | 0 | H |
| 16 | -8.791 | -0.586 | -9.377 | 6 | 14 | H |
| 17 | -8.717 | -0.554 | -9.271 | 0 | 0 | H |
| 18 | -8.407 | -0.698 | -9.105 | 1 | 0 | H |
| 19 | -8.278 | -0.879 | -9.157 | 38 | 0 | H |

**Table 5.** Hydration sites analysis of PPARγ. Each hydration sites were numbered from the highest to lowest count of oxygen atom of water. The enthalpic and entropic energies of each hydration sites and the sum of both energies, E_TOT, were presented. The ratios of hydrogen bonding donors and acceptors are also presented. In addition, pharmacophore feature assigned by our scheme is presented and the selected feature for the model is highlighted by yellow shading.

| **Number** | **Enthalpy**  **(kcal/mol)** | **Entropy**  **(kcal/mol)** | **E_TOT**  **(kcal/mol)** | **%DONOR** | **%ACCEPTOR** | **Pharmacophore feature** |
| --- | --- | --- | --- | --- | --- | --- |
| 1 | -9.771 | -4.086 | -13.857 | 162 | 1 | D |
| 2 | -9.716 | -3.431 | -13.148 | 85 | 0 | - |
| 3 | -8.855 | -3.368 | -12.223 | 85 | 42 | - |
| 4 | -11.300 | -2.738 | -14.038 | 0 | 165 | N |
| 5 | -9.776 | -2.683 | -12.459 | 0 | 0 | - |
| 6 | -9.052 | -2.543 | -11.596 | 1 | 0 | - |
| 7 | -9.833 | -2.677 | -12.510 | 8 | 2 | - |
| 8 | -10.263 | -2.334 | -12.597 | 0 | 92 | - |
| 9 | -10.817 | -2.251 | -13.068 | 0 | 85 | - |
| 10 | -11.060 | -2.858 | -13.918 | 60 | 69 | N |
| 11 | -10.674 | -2.885 | -13.558 | 0 | 91 | - |
| 12 | -8.105 | -1.982 | -10.087 | 54 | 1 | - |
| 13 | -10.066 | -2.269 | -12.335 | 122 | 23 | D |
| 14 | -6.830 | -2.335 | -9.165 | 77 | 0 | - |
| 15 | -9.969 | -2.493 | -12.461 | 46 | 0 | - |
| 16 | -9.657 | -2.263 | -11.920 | 0 | 1 | - |
| 17 | -8.935 | -1.418 | -10.353 | 15 | 1 | H |
| 18 | -10.336 | -1.680 | -12.016 | 1 | 0 | - |
| 19 | -9.363 | -1.324 | -10.687 | 10 | 0 | H |
| 20 | -8.720 | -0.813 | -9.533 | 13 | 1 | H |
| 21 | -8.743 | -0.770 | -9.512 | 3 | 0 | H |
| 22 | -8.765 | -1.626 | -10.390 | 1 | 0 | H |
| 23 | -9.492 | -0.969 | -10.461 | 1 | 1 | H |
| 24 | -8.511 | -0.803 | -9.314 | 0 | 0 | - |
| 25 | -10.164 | -2.023 | -12.187 | 0 | 4 | - |

**Table 6.** Hydration sites analysis of PARP. Each hydration sites were numbered from the highest to lowest count of oxygen atom of water. The enthalpic and entropic energies of each hydration sites and the sum of both energies, E_TOT, were presented. The ratios of hydrogen bonding donors and acceptors are also presented. In addition, pharmacophore feature assigned by our scheme is presented and the selected feature for the model is highlighted by yellow shading.

| **Number** | **Enthalpy**  **(kcal/mol)** | **Entropy**  **(kcal/mol)** | **E_TOT**  **(kcal/mol)** | **%DONOR** | **%ACCEPTOR** | **Pharmacophore feature** |
| --- | --- | --- | --- | --- | --- | --- |
| 1 | -10.719 | -4.105 | -14.825 | 107 | 108 | - |
| 2 | -10.355 | -3.659 | -14.014 | 92 | 1 | - |
| 3 | -9.814 | -2.357 | -12.171 | 1 | 81 | - |
| 4 | -9.859 | -2.645 | -12.504 | 103 | 17 | D |
| 5 | -11.009 | -2.688 | -13.697 | 95 | 0 | - |
| 6 | -10.522 | -2.395 | -12.917 | 81 | 0 | - |
| 7 | -9.796 | -2.802 | -12.598 | 0 | 118 | A |
| 8 | -9.197 | -2.120 | -11.317 | 77 | 0 | - |
| 9 | -9.655 | -1.683 | -11.339 | 0 | 80 | - |
| 10 | -11.351 | -2.906 | -14.257 | 94 | 100 | - |
| 11 | -9.690 | -2.029 | -11.719 | 93 | 0 | - |
| 12 | -9.854 | -2.116 | -11.970 | 83 | 22 | - |
| 13 | -10.799 | -2.184 | -12.983 | 90 | 0 | - |
| 14 | -9.884 | -1.582 | -11.466 | 0 | 85 | - |
| 15 | -10.335 | -1.842 | -12.177 | 42 | 0 | - |
| 16 | -9.469 | -1.591 | -11.059 | 0 | 0 | H |
| 17 | -10.703 | -2.230 | -12.932 | 93 | 0 | - |
| 18 | -9.528 | -1.902 | -11.430 | 53 | 0 | - |
| 19 | -9.484 | -1.777 | -11.262 | 69 | 0 | - |
| 20 | -9.921 | -1.795 | -11.717 | 108 | 0 | D |
| 21 | -10.335 | -1.591 | -11.926 | 48 | 0 | - |
| 22 | -9.852 | -1.026 | -10.878 | 0 | 0 | - |
| 23 | -9.553 | -0.887 | -10.440 | 0 | 0 | - |
| 24 | -10.966 | -2.148 | -13.114 | 133 | 0 | D |
| 25 | -10.486 | -1.778 | -12.264 | 79 | 0 | - |
| 26 | -9.882 | -1.606 | -11.488 | 1 | 10 | - |
| 27 | -9.682 | -1.219 | -10.901 | 18 | 1 | - |
| 28 | -9.736 | -1.355 | -11.091 | 0 | 0 | - |
| 29 | -8.407 | -1.191 | -9.599 | 39 | 0 | - |
| 30 | -9.222 | -0.844 | -10.065 | 0 | 0 | H |
| 31 | -9.524 | -0.771 | -10.294 | 1 | 0 | - |
| 32 | -9.654 | -0.626 | -10.280 | 2 | 0 | - |
| 33 | -9.734 | -0.641 | -10.375 | 0 | 0 | - |
| 34 | -9.858 | -0.676 | -10.535 | 0 | 0 | - |
| 35 | -9.399 | -0.352 | -9.751 | 6 | 0 | R |
| 36 | -9.817 | -0.669 | -10.485 | 0 | 0 | - |

**Table 7.** Hydration sites analysis of AChE. Each hydration sites were numbered from the highest to lowest count of oxygen atom of water. The enthalpic and entropic energies of each hydration sites and the sum of both energies, E_TOT, were presented. The ratios of hydrogen bonding donors and acceptors are also presented. In addition, pharmacophore feature assigned by our scheme is presented and the selected feature for the model is highlighted by yellow shading.

| **Number** | **Enthalpy**  **(kcal/mol)** | **Entropy**  **(kcal/mol)** | **E_TOT**  **(kcal/mol)** | **%DONOR** | **%ACCEPTOR** | **Pharmacophore feature** |
| --- | --- | --- | --- | --- | --- | --- |
| 1 | -10.583 | -4.196 | -14.780 | 90 | 98 | - |
| 2 | -9.716 | -5.264 | -14.981 | 131 | 87 | - |
| 3 | -13.872 | -5.032 | -18.904 | 192 | 0 | D |
| 4 | -11.104 | -3.905 | -15.009 | 93 | 51 | - |
| 5 | -11.416 | -4.374 | -15.790 | 85 | 0 | - |
| 6 | -9.289 | -2.430 | -11.719 | 0 | 0 | - |
| 7 | -10.979 | -3.133 | -14.112 | 5 | 94 | - |
| 8 | -10.254 | -2.590 | -12.843 | 0 | 0 | - |
| 9 | -8.745 | -2.942 | -11.686 | 79 | 69 | - |
| 10 | -10.227 | -2.907 | -13.134 | 41 | 0 | - |
| 11 | -10.075 | -2.797 | -12.872 | 0 | 91 | - |
| 12 | -10.768 | -2.283 | -13.051 | 1 | 0 | - |
| 13 | -10.437 | -2.713 | -13.150 | 78 | 0 | - |
| 14 | -10.917 | -2.618 | -13.535 | 88 | 0 | - |
| 15 | -8.303 | -2.456 | -10.759 | 0 | 0 | - |
| 16 | -10.719 | -2.391 | -13.110 | 6 | 0 | - |
| 17 | -10.257 | -2.940 | -13.197 | 75 | 155 | - |
| 18 | -10.526 | -2.112 | -12.637 | 56 | 8 | - |
| 19 | -9.782 | -2.243 | -12.025 | 95 | 0 | - |
| 20 | -8.912 | -2.460 | -11.373 | 90 | 0 | - |
| 21 | -9.919 | -2.046 | -11.965 | 48 | 0 | - |
| 22 | -9.567 | -1.546 | -11.113 | 38 | 0 | - |
| 23 | -10.116 | -1.181 | -11.297 | 0 | 93 | - |
| 24 | -10.066 | -1.808 | -11.874 | 96 | 0 | - |
| 25 | -9.805 | -1.881 | -11.686 | 115 | 0 | D |
| 26 | -10.277 | -1.870 | -12.147 | 47 | 0 | - |
| 27 | -10.003 | -1.973 | -11.975 | 2 | 0 | - |
| 28 | -10.034 | -1.825 | -11.858 | 72 | 61 | - |
| 29 | -10.262 | -1.610 | -11.871 | 94 | 0 | - |
| 30 | -9.566 | -1.152 | -10.718 | 13 | 73 | - |
| 31 | -10.070 | -1.802 | -11.872 | 78 | 0 | - |
| 32 | -9.439 | -0.869 | -10.308 | 42 | 88 | H |
| 33 | -9.790 | -1.423 | -11.213 | 0 | 0 | - |
| 34 | -9.219 | -1.116 | -10.335 | 1 | 0 | H |
| 35 | -9.327 | -1.038 | -10.366 | 48 | 0 | H |
| 36 | -10.324 | -1.352 | -11.676 | 0 | 0 | - |
| 37 | -9.634 | -0.972 | -10.606 | 0 | 0 | - |
| 38 | -9.569 | -0.936 | -10.505 | 2 | 0 | - |
| 39 | -9.577 | -1.850 | -11.427 | 115 | 0 | D |
| 40 | -9.164 | -0.618 | -9.782 | 46 | 0 | H |
| 41 | -9.615 | -1.313 | -10.928 | 71 | 1 | - |
| 42 | -9.579 | -1.183 | -10.762 | 54 | 0 | - |
| 43 | -9.345 | -1.226 | -10.572 | 86 | 1 | H |
